# Reversible Binding Interfaces Made of Microstructured Polymer Brushes

**DOI:** 10.1021/acs.langmuir.4c00062

**Published:** 2024-03-25

**Authors:** Ronaldo Badenhorst, Sergei Makaev, Dmytro Yaremchuk, Yash Sajjan, Artem Sulimov, Vladimir V. Reukov, Nickolay V. Lavrik, Jaroslav Ilnytskyi, Sergiy Minko

**Affiliations:** † Nanostructured Material Lab, University of Georgia, Athens, Georgia 30602, United States; ‡ Institute for Condensed Matter Physics of the National Academy of Sciences of Ukraine, Lviv, 790011, Ukraine; § Department of Chemistry, University of Georgia, Athens, Georgia 30602, United States; ∥ Department of Textiles, Merchandising, and Interiors, University of Georgia, Athens, Georgia 30602, United States; ⊥ Center for Nanophase Materials Sciences, Oak Ridge National Lab, Oak Ridge, Tennessee 37831, United States; # Institute of Applied Mathematics and Fundamental Sciences, Lviv Polytechnic National University, Lviv, UA-79013, Ukraine

## Abstract

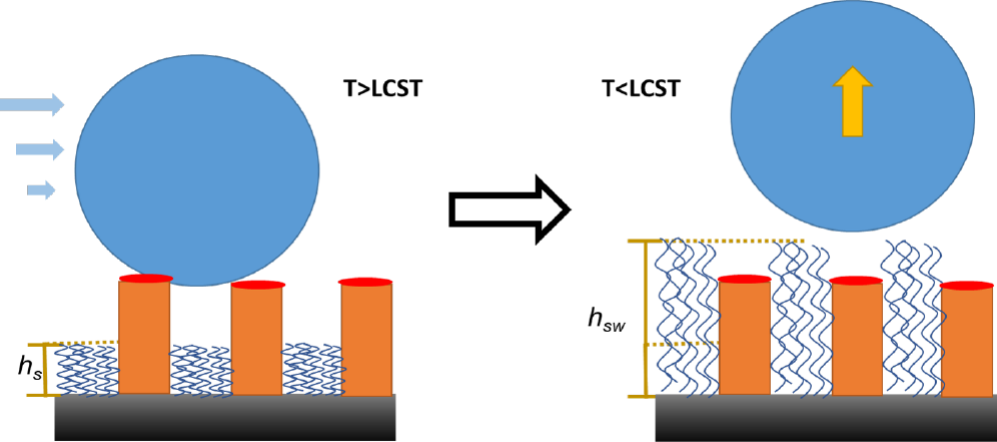

The polymer brush architecture of the end-tethered polymer molecules is one of the most widely used efficient methods to regulate interfacial interactions in colloidal systems found in live matter and manufactured materials. Emerging applications of polymer brush structures require solutions to new tasks in the control of interfacial interactions. The rapid development of live cell manufacturing relies on scalable and efficient cell harvesting methods. Stimuli-responsive surfaces made of surface-grafted poly(*N*-isopropylacrylamide) (PNIPAM) can bind and detach the adherent cell upon changes in temperature and have been used for cell growth and harvesting. The applications are limited by the requirement to satisfy a range of PNIPAM coating characteristics that depend on the dimensions of the integrin complex in the cell membrane and the basal surface. The analysis of the microstructured surfaces, when adhesive and disjoining functions of the microdomains are decoupled, shows that many limitations of PNIPAM one-component coatings can be avoided by using a much broader range of structural characteristics of the microstructured interfaces composed of alternating disjoining PNIPAM domains and adhesive polymeric domains with cell-affinity functional groups. Temperature-controlled reversible adhesion to such microstructured interfaces is studied here experimentally with model systems of solid spherical particles and by employing simulations for solid and soft membranes interacting with the microstructured surfaces to mimic interactions with soft and solid disk-like particles.

## Introduction

Reversible binding between different objects is realized when the thermal energy is comparable to the energy of intermolecular interactions, when binding occurs at low temperatures, and when detachment occurs at high temperatures. This mechanism is observed for the reversible adsorption of small molecules and is practically used in separation technologies and chromatographic analysis. However, for many applications with colloidal objects, due to the high interfacial contact area, the latter scenario would require substantial changes in temperature, which is impractical or impossible. The most realistic approach is to use minor changes in temperature or other properties of the system depending on the composition of the environment of the adhesive joint to weaken intermolecular interactions or even generate electrostatic and steric repulsion between the objects. The latter scenario requires a specially designed interface located between the adhered objects.

Reversible adsorption, adhesion, or binding-detachment of colloidal particles at the liquid–solid interface have been associated with stimuli-responsive interfaces.^1^ For simplicity, in the contents of this article, we call such interfaces “reversible interfaces.″ They are usually made of one or two polymers grafted onto the solid substrate. In the case of a single polymer, the binding-detachment reversible process is realized through changes in the polymer–liquid interaction, which typically involves changes in the phase behavior and chemical equilibrium. The same mechanisms are involved in the reversible changes of the polymer interfaces made of two or even more mixed polymers, but in this case, the phase behavior is strongly modified by polymer–polymer interactions.^2,3^ Changes in pH achieve the most remarkable reversible changes in interfacial properties if the polymer at interfaces is a weak polyelectrolyte.^4^ In the latter case, the involvement of electrostatic interactions allows for switching from attractive to repulsive interactions between the interface and colloidal particles.

Besides interesting basic science research to understand the behavior of stimuli-responsive interfaces, the reversible interfaces were demonstrated to find practical applications such as self-cleaning surfaces,^5,6^ reversible colloidal stability,^7,8^ capture and release of proteins, DNA, and different microscopic objects for drug delivery applications,^9^ sensors,^10^ and increased packing of colloids in dense particle monolayers for optical applications.^11^ Most such developments remained at the proof of concept stage, mainly because typical real-life applications of stimuli-responsive interfaces require an additional combination of properties that may not be compatible with stimuli-responsive behavior, and also the design of such interfaces can be prohibitively expensive for many applications.

Here, we are interested in the potential application of stimuli-responsive interfaces for live cell manufacturing technologies. The rapid development of biotechnology that employs live cells faces technical problems of cell production at industrial scales. One of the critical step of adherent cell manufacturing is cell harvesting. Commonly used, enzymatic cell detachment is less desired for many applications because of cell damage that becomes critical for scaling up.^12−16^

**Figure 1:**
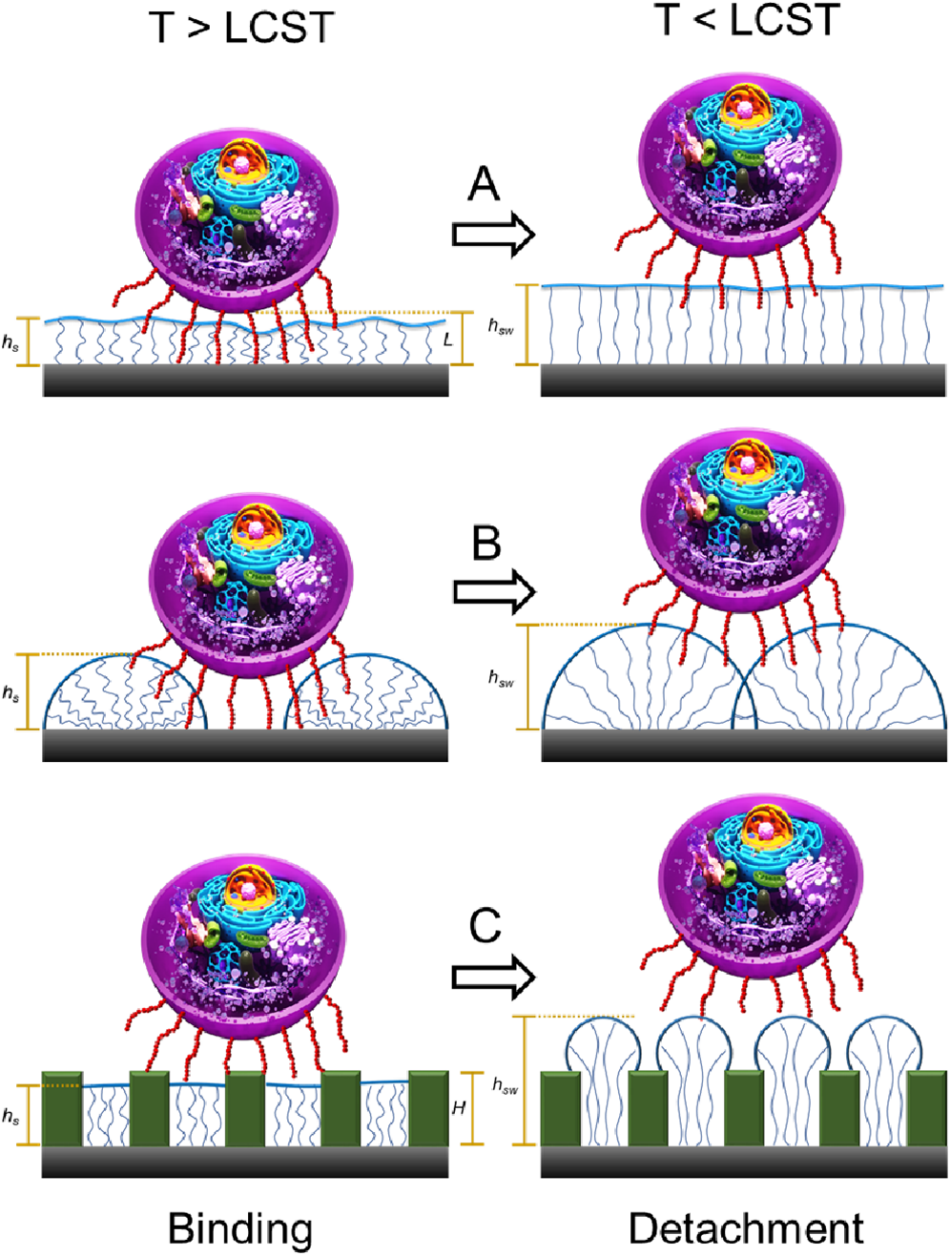
Schematics of reversible binding to the PNIPAM brushes of different designs: A-uniform PNIPAM layer; B-patterned brush; C-patterned brush with asperities of the height *H*; *h*_*s*_ – is the brush thickness above LCST and *h*_sw_ – is the brush thickness below LCST.

Poly(*N*-isopropylacrylamide) (PNIPAM) thermoresponsive interfaces have been introduced by Okano et al.^17^ and other teams^18−23^ as an alternative to enzymatic cell harvesting. A PNIPAM polymer brush-like layer or a cross-linked PNIPAM film is typically grafted to the solid support (glass or polystyrene) on and from which cell attachment and detachment is required. By changing the cell culture temperature from 37 °C to a temperature below 32 °C (lower critical solution temperature, LCST, for PNIPAM in aqueous solution), swelling of the PNIPAM film leads to single cell or cell sheet detachment (Figure 1A). However, many publications reported inconsistency in the performance of such surfaces when either poor adhesion or poor detachment was observed. The type of cells, the kind of basal supporting material, and the PNIPAM layer thickness affected the latter. Halperin et al.^24^ explained such inconsistency by a mismatch between the shrunken PNIPAM layer thickness and the size of the integrin binding protein complex (*L*) – the major mechanism of binding for adherent cells. There are two related characteristic thicknesses of the PNIPAM layer: the shrunken layer thickness *h*_s_ is the thickness above LCST, and the swollen layer thickness *h*_*sw*_ is the thickness below LCST. Their combination affects cell binding and detachment. The cell binding is efficient if *L* > *h*_s_ while the detachment is efficient if *L* < *h*_sw_.

The integrin complex cannot bind directly to PNIPAM, but the binding is realized through the adsorption of extracellular matrix (ECM) proteins that carry integrin-selective motifs. The characteristic length *L*, including those of ECM proteins, is about 20–25 nm. The PNIPAM layer is characterized by two ratios: the swelling ratio above LCST *R*_A_ = *h*_s_*/h* and the swelling ratio below LCST *R*_*B*_ = *h*_sw_*/h*, where *h* is the PNIPAM dry layer thickness. Consequently, the conditions of reversible binding can be expressed as *L*/*R*_B_ < *h* < *L*/*R*_A_. These conditions depend on the dimensions of the binding complex and the swelling degree of the PNIPAM layer. The shrunk PNIPAM layer contains about 30–40% water above LCST, while depending on the molecular characteristics, PNIPAM layers swell 1.5–4 times below LCST. For approximate estimation, we can choose *R*_A_ = 1.35 and *R*_B_ = 3; consequently, 8 nm < *h* < 19 nm. That leaves a relatively narrow window of about 11 nm for the PNIPAM layer thickness to provide the combination of binding and detachment properties. This limitation is because the same material binds and detaches cells.

The mechanism of reversible adhesion shown in Figure 1A can be extended to the general case of binding of various colloidal particles when binding sites are randomly distributed in the stimuli-responsive film. In this case, the particle detachment can be achieved if the average distance between the binding sites at a temperature below LCST is greater than that at *T* > LCST. This mechanism introduces another limitation for the structure of stimuli-responsive layers, defined as a change in the distance between adhesive points upon swelling.

López et al.^25^ proposed a different architecture for reversible binding. Suppose the PNIPAM brush is grafted on surface patterns. In that case, the distance between patterns can be adjusted to switch between two states: the complete coverage of the surface by the brush below LCST and only partial coverage of the surface by changing temperature to *T* > LCST (Figure 1B). However, the same condition 8 nm < *h* < 19 nm is applied. In this case, the swelling degree of the brush is lower than that for the brush uniform layer so that the range for *h* can be even narrower. However, separating the adhesive and disjoining domains brings benefits to controlling both binding and detachment independently.

Recently, we developed an alternative design for the patterned polymer brush with a PNIPAM brush grafted between an array of pillars that serve as adhesive domains (Figure 1C), while the PNIPAM brush provides the disjoining mechanism.^26^ For such architecture of the interface, the conditions for a combination of binding and detachment can be expressed as (*H* + *L*)*/R*_B_ < *h < H*/*R*_A_ or for *H* ≫ *L*, 1*/R*_B_ < *h/H<*1/*R*_A_. Practically, it is possible to eliminate the dependence of the reversible binding on the size of the binding protein complex when the ratio of the brush to asperity (adhesive domain) height defines the performance of the reversible interfaces. Consequently, we can speculate that this method can be applied to various types of live cells and colloidal particles with different binding mechanisms and that the binding and disjoining energies can be adjusted independently.

In this work, we are making the next steps to understanding the mechanism of reversible binding to the interfaces that combine patterned brushes and arrays of microasperities by testing them with a model system of colloidal particles. We focus on interfacial phenomena and explore the colloidal particles to exclude biological aspects of the live cell behavior when the latter changes with time of the cell-surface interaction. The reversible binding is analyzed using a model system of colloidal particles with adhesion to the stimuli-responsive interfaces in the range typically observed for live cells bound to the substrates with a low surface concentration of adhesive biomolecules, as estimated by the wall shear stress required to detach particles or cells (typically ranging from 1 to 10 Pa). The analysis is performed by combining experiments and computer simulations.

## Experimental Section

### Materials and Chemicals

Si-wafers were purchased from University Wafer, Boston, MA, USA. (3-aminopropyl) triethoxysilane (APTES), polystyrene (PS) microbeads decorated with sulfo groups (AmberChrom 50Wx2 200–400) (ThermoFisher), ascorbic acid (ASCO), α-bromoisobutyryl bromide (BIBB), triethylamine (TEA), copper(II) bromide (CuBr_2_), *N*, *N*, *N*′, *N*″, *N″*-pentamethyl diethylenetriamine (PMDTA), ammonia 25% solution, polyethylenimine (PEI) (*M*_n_ = 10000 g/mol, *M*_w_ = 25000 g/mol), and cyclopentanone were purchased from Millipore Sigma and used as received. Sulfuric acid, dichloromethane, and 30% hydrogen peroxide (H_2_O_2_) were purchased from Fisher Scientific and used as received. *N*-isopropylacrylamide (97%) (NIPAM) was purchased from Millipore Sigma and recrystallized in hexane to remove inhibitors prior to polymerization. An epoxy-based photoresist SU-8 2002 and the SU-8 developer were purchased from Kayaku Advanced Materials, Inc.

### Fabrication of the Photomask

GDSII files of the patterns were compiled using the LayoutEditor software (https://layouteditor.com/). The compiled GDSII files were used to write patterns on 5 in. glass photomasks with Cr metalization on the Heidelberg DWL66 tool (Heidelberg-Instruments, Gmbh). Masks with exposed photoresist were developed in the CD-26 developer, rinsed in deionized water, dried, and subsequently etched in acid–based the Cr-14 chromium etchant for 2 min. After that, the remaining photoresist was removed in hot *N*-methyl-2-pyrrolidone, rinsed with deionized water, and dried.

### Functionalization of the Surface of Si-Wafers with the Initiator

After cutting Si-wafers into 0.9 × 0.9 cm^2^ square samples, they were cleaned in Piranha solution (1:1:1 ratio of ammonium hydroxide, deionized (DI) water, and H_2_O_2_, alternatively a 3:1 ratio of concentrated sulfuric acid and H_2_O_2_) for 60 min at 70 °C. Warning! Piranha solutions are highly reactive oxidizing reagents and are not stable. They should be used with care and personal protection measures. The cleaned samples were rinsed with DI water and ethanol, and dried under an argon flux. After washing and cleaning the Si-wafers, they were immersed in a 1% APTES solution in toluene for 1 h to functionalize the surface with amino groups for further functionalization. The surface-bound atom transfer radical polymerization (ATRP) initiator for the NIPAM polymerization was immobilized by immersion of the amino-functionalized Si-wafers in 1% TEA and 2% BIBB in anhydrous chloroform for 3 h at room temperature. The latter step was followed by rinsing with ethanol and drying under argon. The functionalized Si-wafers were then stored in sealed containers until they were used, preferably within 24 h.

### Fabrication of SU-8 Structures Using Lithography

Before grafting the PNIPAM brush, the cross-linked SU-8 photoresist microstructure was fabricated using photolithography to form adhesive domains expected to bind particles. A dynamic dispense spin-coating was conducted at 4000 rpm for 40 s, using SU-8 2002 (diluted with cyclopentanone to 10% solution) on the initiator functionalized Si-wafer samples. The formed SU-8 films were baked at 90 °C for 1 min and then exposed to UV light (365 nm, 7 mW/cm^2^) for 30 s through the photomask. A postexposure bake of the samples was carried out on a hot plate at 90 °C for 1 min. The Si-wafers were immersed into the SU-8 developer for 1 min, followed by a 10 s immersion in isopropanol, drying under an argon flux and a hard bake at 150 °C for 5 min.

### Grafting of the PNIPAM Brush

Graft polymerization of NIPAM on the samples with the developed SU-8 structures was carried out via the ARGET-ATRP mechanism as reported elsewhere.^27^ NIPAM was grafted only from the area between SU-8 domains using the surface-bound initiator unmasked after development of the photoresist. The sample was immersed in 1200 μL of a 50 wt % NIPAM solution in a 2:1 methanol:water mixture. Then 95 μL of 0.05 g/mL CuBr_2_ in water and 150 μL of 0.05 g/mL PMDTA water were added in a vial closed with a rubber septum. Oxygen was removed by purging the solution with argon for 30 min. Then, 100 μL of ASCO (0.05 g/mL in water) was added into the polymerization vial through the septum; the vial was sealed afterward. The polymerization was conducted at room temperature for 20, 40, and 60 min and longer. The polymerization was stopped by opening the cap, and the sample was rinsed with ethanol to remove residual monomers, bulk polymers, and other chemicals. The samples with uniform PNIPAM coatings (no photoresist structures) for control experiments were prepared by grafting from the ATRP initiator using the same procedures for the initiator immobilization and grafting of NIPAM as described above. The molecular mass of grafted PNIPAM was estimated by the measurements of the molecular mass of the bulk polymer using the static light scattering method (Table 1). The estimated grafting density based on the molecular mass and dry thickness of the brush for all the synthesized samples was 0.2 nm^–2^ (the standard deviation 0.05 nm^–2^).

**Table 1:** Samples of Patterned Brushes: The Rectangular Domains (Pillars) are Made of SU-8, While the Interpillar Space is Decorated with PNIPAM Brushes^a^

		height, nm						
sample, *M*_w_, kg/mol	SU-8 size,^b^ D, μm^2^ (space, μm)	SU-8, *H*	PNIPAM, *h*	*h*/*H*	*R*_B_ = *h*_sw_/*h*	1/*R*_B_	*G*_A_^c^	*G*_B_^d^
S1 253	4 × 4 (3)	59.0 (5.5)	76.4 (4.1)	1.30 (0.12)	2.43 (0.3)	0.41 (0.05)	1.53 (0.05)	0.83 (0.08)
S2 266	4 × 4 (3)	37.2 (4.1)	80.2 (0.4)	2.18 (0.23)	3.17 (0.1)	0.32 (0.01)	1.94 (0.01)	0.60 (0.04)
S3 159	4 × 4 (3)	57.2 (7.4)	48.0 (1.9)	0.85 (0.09)	4.32 (0.2)	0.23 (0.01)	1.49 (0.01)	0.76 (0.03)
S5 131	5 × 5 (3)	69.5 (3.5)	39.5 (3.5)	0.57 (0.2)	4.06 (0.1)	0.26 (0.01)	1.80 (0.01)	0.48 (0.12)
S6 73.5	3.5 × 3.5 (2.5)	90.7 (8.6)	22.2 (6.0)	0.24 (0.05)	4.63 (0.5)	0.22 (0.02)	1.15 (0.03)	0.44 (0.06)
S7 158	3.5 × 3.5 (2.5)	42.3 (2.1)	47.7 (1.2)	1.13 (0.08)	2.87 (0.4)	0.35 (0.05)	1.61 (0.05)	0.69 (0.08)
S8 216	4 × 4 (3)	53.5 (8.6)	65.3 (7.3)	1.23 (0.15)	2.71 (0.4)	0.38 (0.06)	1.65 (0.06)	0.74 (0.11)
S9 215	4 × 4 (3)	52.5 (2.4)	64.8 (4.7)	1.24 (0.14)	2.70 (0.1)	0.37 (0.02)	1.57 (0.02)	0.73 (0.04)

a Standard deviations are given in brackets.

b Photomask dimensions

c

d , where *R*_*A*_ = *h*_*s*_*/h≈*1.35 is the swelling of PNIPAM at *T* > LCST, *R* is the radius of the adsorbed particle.

### Functionalization with PEI

**Figure 2:**
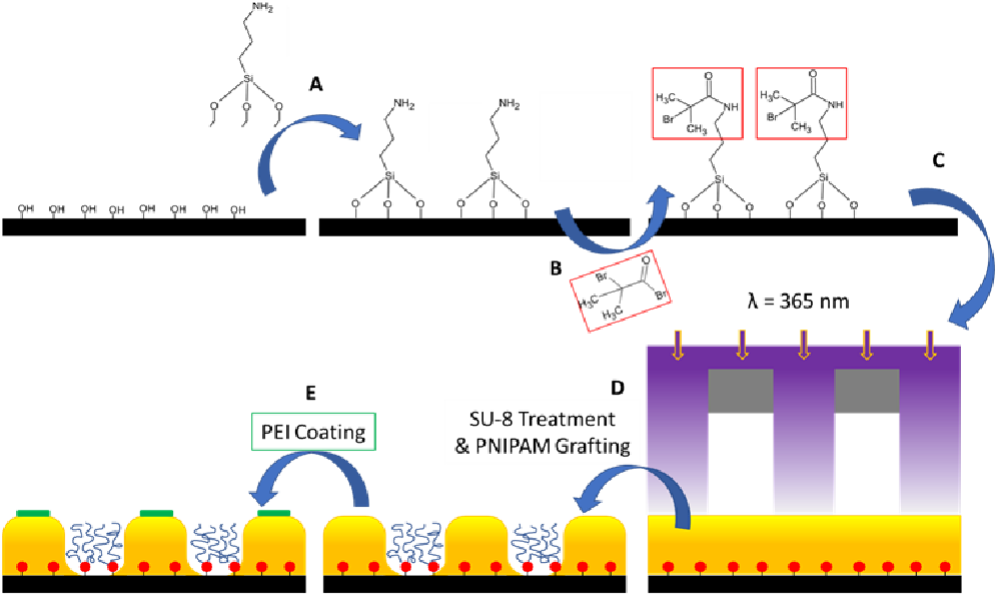
Cut, cleaned, and Piranha-treated Si-wafer was functionalized with APTES in toluene (A), which, after washing and drying, was treated with BIBB in CHCl_3_ (B) to complete the immobilization of the ATRP initiator onto the wafer surface. Prior to polymerization, the initiator-functionalized Si-wafer was decorated with SU-8 microstructured domains using photolithography and photomasks (C). Following the formation of the domains, treatment included soft baking at 90 °C, removal of untreated SU-8 with a developer solution, and hard baking at 150 °C, whereafter PNIPAM grafting was initiated on the still exposed Si-wafer surfaces (D). Selective coverage of SU-8 domains with PEI was achieved by spin coating the entire surface with PEI, followed by covalent bonding initiated by heating at 150 °C, and the unbound PEI was removed by a solution in water and acidic water washing steps (E).

Branched PEI was used to introduce a positive surface charge on the surface of the SU-8 domains by grafting PEI to the residual epoxy groups of SU-8. A solution of 0.1% PEI in EtOH was prepared and applied to SU-8 and PNIPAM decorated Si-wafers by spin coating at 4000 rpm with an acceleration of 500 rpm/s for 40 s on the preheated samples at 80 °C for 1 min. Following the coating step, the coated Si-wafers were baked at 150 °C for 5 min and placed in DI water for 1 h, followed by a pH 2 aqueous HCl solution for 3 h, after which the Si-wafers were dried and stored. The deposition process was optimized on single component control films made from SU-8 and PNIPAM, respectively. Ellipsometry demonstrated that 4 mg/m^2^ of PEI was surface-grafted to SU-8 films, while the described protocol allowed the washing out of all PEI from the PNIPAM surface. The schematic for the fabrication of the reversible interfaces is shown in Figure 2.

### Characterization of the Microstructured Films

Microstructured polymer films were characterized by scanning probe microscopy (SPM); a Bruker Dimension Icon microscope was used to measure the domain size structural changes in dry (air, 25 °C) and aqueous states (water, 25 °C), along with a Bruker Multimode 8 for similar measurements in the aqueous state at 37 °C. For Dimension Icon, the samples (∼10 × 10 mm^2^) were placed on the sample support table and held in place by vacuum. For the Multimode 8 measurements, the samples (∼10 × 10 mm^2^) were glued to a stainless-steel disk, which was magnetically fixed to the Multimode heater/cooler accessory, which in turn was magnetically fixed to the scanner head. Measurements were conducted in the Bruker patented PeakForce Tapping mode in air and in liquid. For air measurements, Bruker RTESPA-300 probes (rectangular probe, nominal resonance frequency 300 kHz, spring constant 40 N/m, tip radius 8 nm, aluminum reflective coating) were used. For liquid measurements, NanoWorld PNP-Tr-Au probes (triangular probe, nominal resonance frequency in air of 17 kHz, spring constant of 0.08 N/m, tip radius of 40 nm, gold reflective coating) were used.

Polymer films were scratched with a razor blade to obtain the basal surface level (silicon substrate) to serve as a reference for height measurement. The scan resolution was fixed at 512 × 512 pixels; the scan areas varied from 8 × 8 to 16 × 16 μm^2^. The collected scans were leveled using the Gwyddion software and exported as 16-bit grayscale images to Fiji software to measure the domain area and perimeter using threshold analysis. Each sample was scanned in several (2–7) locations, and the results were averaged.

The thicknesses of one component (grafted PNIPAM or spin-coated SU-8) films of the control samples were estimated with ellipsometry using a variable angle Accurion Nanofilm EP4 ellipsometer at a fixed wavelength of 658 nm. The scan area varied from 300 × 300 to 1000 × 1000 μm^2^. The difference between SPM and ellipsometry thickness measurements was within 5–10%. Microstructured surfaces were not measured using ellipsometry because the scan area is much greater than the size of the domains.

Scanning electron microscopy (SEM) imaging of the microstructured surfaces was conducted using the Thermo Fisher Scientific (FEI) Teneo microscope at a 2 kV accelerating voltage and 2000–5000× magnification.

### Cell Culture Preparation

RAW 264.7 (ATCC TIB-71) macrophages were purchased from ATCC (Manassas, VA), and the NIH3T3/GFP fibroblast cell line was generously donated by BioAesthetics Corp. (Durham, NC). Cell lines were cultured with Dulbecco’s Modified Eagle’s Medium (DMEM, Corning), supplemented with 200 U/mL penicillin, 200 mg/mL streptomycin, and 10% fetal bovine serum (Millipore-Sigma). The cells were incubated at 37 °C and 5% CO_2_ and subcultured at about 80% confluency.

### Cell Binding and Proliferation on the Reversible Interfaces

RAW 264.7 macrophages were grown on the samples of the thermoresponsive coatings. The samples of the culture support materials were placed in a sterile 24-well plate, and then the cells were seeded at a density of 2.5 × 10^4^ cells per well with 1 mL supplemented DMEM. After overnight incubation, the cells were rinsed with warm phosphate-buffered saline (Corning), and fresh medium was added, supplemented with 0.1 μg/L lipopolysaccharide (Millipore-Sigma) to induce hyperadhesion response. Cells were incubated for 24h in a 5% CO_2_ humidified atmosphere incubator at 37 °C before testing. The surface-bound cells were contrasted with Calcein AM, actin staining Phalloidin, and 4′,6-diamidino-2-phenylindole (Millipore-Sigma), and visualized using an EVOS M5000 fluorescence microscope.

### Characterization of Polystyrene Beads

Polystyrene-based ion-exchange resin AmberChrom 50Wx2 beads, hydrogen form, 200–400 mesh size (Thermo Scientific) was used without any pretreatment. The beads were dispersed in a NaOH aqueous solution (∼1% concentration), and the droplet of the colloidal dispersion was placed on the Si-wafers. After adsorption of the particles in 3 min, the images were taken using an Olympus BX51 microscope with a Tucsen TCC-3.3ICE-N camera. The collected images were converted to 16-bit grayscale in Fiji software; the size, number, and distribution of beads by size were obtained using threshold analysis. The optical images of the PS beads are shown in Figure S1; the estimated average radius is 37.4 μm (Figure S1).

### Reversible Adhesion Tests

Prior to testing, feed NaOH solutions in DI water were adjusted to pH 9–9.5. The feed solution was circulated through the piping and flow cell to achieve an adequately heated and cooled flow cell before testing. The Si-wafers were placed and held in place by double-sided tape within the flow cell. Prior to closing the flow cell for testing, the PS microbeads in a DI water dispersion were placed on the surface of the microstructured interface using a pipette dropper. The solution was spread across the entire surface, and the flow cell was closed and sealed with screws to prevent leakage. Testing was done by adjusting the flow rate to set points for 3 min, after which a picture of the sample’s surface was taken, and then the flow rate was increased to the next point. The images for the analysis were taken with an optical microscope set at ×5 magnification. The image analysis was performed to estimate the number of PS beads on the surface and the surface coverage by the beads at different shear stresses generated by the liquid flux. The shear stress was estimated based on the liquid flow rate and geometry of the fluidic channel using the Poiseuille model. The schematic of the fluid cell, the experiment, and the calculation of the shear stress are shown in Figure S2 and Table S1.

### Simulation Approach

Simulations were performed using the dissipative particle dynamics (DPD) method.^28^ We consider the PNIPAM polymer and water first. Each PNIPAM chain comprises an array of bonded soft-core spherical beads, whereas water is modeled as a set of separate beads. The diameters of both polymer and water beads, σ, their masses, *m*, the energy scale *k*_B_*T* (here *k*_B_ is the Boltzmann constant), the temperature, *T,* and the time unit, *t*, are all set as σ = *m* = *k*_B_*T* = *t* = 1.^28^ The magnitude of the repulsive force acting between the ith and jth beads is
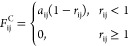
1

where *a*_ij_ is the density and temperature-dependent repulsion strength and *r*_ij_ is the separation between the beads. For the polymer–polymer and water–water interactions, the parametrization by Groot and Warren^28^*a*_ij_ = 25 is typically used. It is obtained for the bulk number density of beads equal to  by matching the model compressibility and that of water under normal conditions.^28^ For the PNIPAM-water interaction, we used the parametrization based on the temperature dependence of the Flory–Huggins mixing parameter χ between PNIPAM and water,^29^ resulting in *a*_ij_ = 25.6 for the swollen state at *T* = 25 °C < LCST, and *a*_ij_ = 38 for the shrunken (collapsed) state at *T* = 37 °C > LCST. This model was validated before for both an isolated PNIPAM chain and for a polymer brush,^30^ focusing on a range of known scaling laws for various size properties in both collapsed and swollen states for the polymer chains of up to 60 monomers.^30^ This study also indicated the presence of an optimal brush grafting density at which the ratio *h*_sw_/*h*_s_ achieves the maximum value. For the polymer chain of 60 monomers, this ratio was found to be around 2.5, which is close to some experimental values.^27^ This parametrization is also used in this study.

Besides repulsive force, the beads are also subjected to the dissipative (friction), , and random, , forces with the respective magnitudes given by^29^
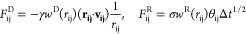
2

where, *v*_ij_ = *v*_i_ – *v*_j_, *v*_i_ and *v*_j_ are respective velocities of the beads, and both magnitudes, σ^2^ = 2γ, and the separation-dependent weight factors, *w*^D^ (*r*_ij_) = *w*^R^ (*r*_ij_)^2^ = (1 – *r*_ij_)^2^, are interrelated.^31^*w*^D^ (*r*_ij_) = (1 – *r*_ij_)^2^ if *r*_ij_ < 1 and is zero otherwise, whereas θ_ij_ is the Gaussian distributed random variable.

The integrity of a polymer chain is achieved by applying the harmonic bonding force with the amplitude
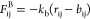
3acting between bonded ith and jth beads, where the choice of *k*_b_ = 4 and *b*_ij_ = 0 is made for the PNIPAM polymer chain. We note that two bonded beads of a polymer chain are not excluded from the pairwise repulsive interaction, and the equilibrium bond length is the result of the competition between these two forces (eq 1 and 3).

The presence of a colloidal particle is reduced to its bottom surface only. On the length scale of the simulation box, it is considered flat. For the sake of brevity, hereafter, we term this surface as a membrane. The presence of the bonds (eq 3) between neighboring beads of a membrane ensures its integrity but does not restrict its folding. We will term this case a *soft membrane*, and it mimics the bottom surface of a cell or an elastic colloidal particle. The other considered case is a *rigid membrane.* The rigidity is introduced via additional bond angles, θ_ijk_, defined between all triplets {*i,j,k*} of beads that are adjacent along the *OY* axis. Bending force amplitude is introduced as
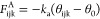
4

where θ_0_ is the reference angle, and the case of θ_0_ = 0 represents an ideal linear arrangement of all three beads. The value of coefficient *k*_a_ defines the level of rigidity of a membrane.

The membrane is made completely transparent for water, *a*_ij_ = 0 for the membrane-water interaction in eq 1, but strongly repulsive for the membrane-polymer pairs, *a*_ij_ = 38. In this way, the osmotic pressure imposed by a polymer brush onto the bottom surface of the particle can be monitored via changes in the membrane shape. To account for the mass of the rest of the particle, which does not enter simulations explicitly, we set rather big masses to each of the membrane beads, namely, *m* = 10^5^.

We also note that special care is taken concerning conservation of the total momentum, ***p***_tot_, in the system. It is split into two parts: ***p***_tot_ = ***p***_beads_ + ***p***_wall_, where  is the momentum of a set of *N* beads, and ***p***_wall_ is the momentum acquired by all walls present in the system as the result of elastic collisions of reflected beads, if any. For the same purpose of conservation of the total momentum, we used a different approach for grafting the required beads to specific points in the simulation box. In contrast to most simulation studies on polymer brushes, instead of using the bonding force (eq 3) we set big masses, *m* = 10^7^ to the grafted beads of each polymer chain or selected grafted beads of the membrane. By monitoring the components of the total momentum, ***p***_tot_, throughout the simulations, we found that their conservation is satisfied with the accuracy of at least of 10^–7^. The simulation time step is chosen equal to *Δt* = 0.005.

### Simulation Setup and Its Workflow

The simulation setup in this study is as follows. The simulation box is the size of *L*_*X*_ × *L*_*Y*_ × *L*_*Z*_, where *L*_*X*_ = 15, *L*_*Y*_ = 69, and *L*_*Z*_ = 40. Reflective walls are introduced along the *OY* and *OZ* axes, whereas periodic boundary conditions are applied along the *OX* axis. The polymer brush is grafted to the bottom surface of the simulation box *z* = 0 onto the rectangular area restricted by the intervals of 0 < *x* < *L*_*X*_ and 0 < *y* < *d*, where *d* = 44. In total, *N*_br_ = 396 linear chains are grafted; therefore, the dimensionless grafting density of PNIPAM is ρ_g_*= N*_br_σ^2^/(*L*_*X*_*d*) ≈ 0.6. Each polymer contains 80 beads. The adjacent fragment of an SU-8 pillar is modeled as an inaccessible region of the simulation box separated from the rest of it via two reflective walls. Its bottom rectangular area is restricted by the intervals of 0 < *x* < *L*_*X*_ and *d* < *y* < *L*_*y*_, whereas its side surface is flat and arranged at an angle θ with respect to the *OY* axis. On its top, the pillar is restricted by a flat surface parallel to the *XY* plane located at *z* = *h*_top_.

## Results and Discussion

### Selection of the Fabrication Method

**Figure 3:**
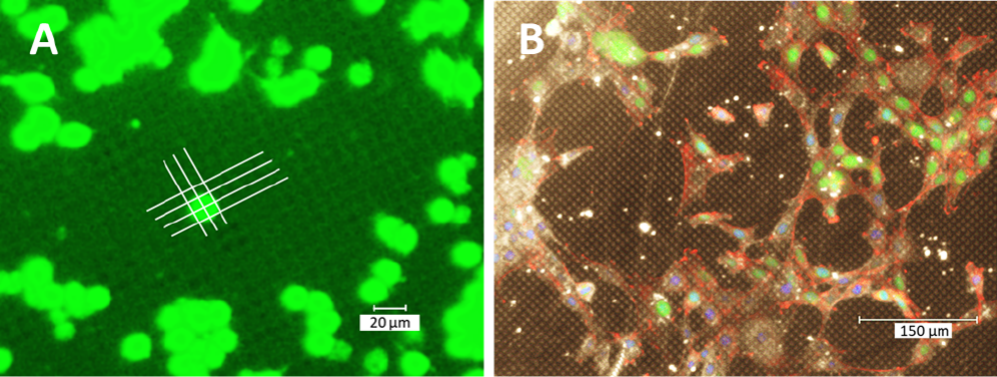
Representative examples of (A) spherical RAW264.7 cells and (B) extended NIH3T3/GFP fibroblast cells on the reversible interface with 4 μm × 4 μm adhesive domains. The white lines are drawn to contrast the structure of the interface. Image A was adapted from ref (26).

When approaching the interface, the typical mammalian cell dimension ranges from 10 to 20 μm. The surface-bound cell can spread over the surface up to a 100 μm elongated shape. The deviation from the spherical shape depends on the cell type and the cell-surface interactions. A live cell is an elastic and changeable structure and can somewhat adapt to the surface topography. The hypothesis is that the surface-bound cell can be detached with minimal damage by applying an oversurface contact area disjoining pressure generated by small disjoining domains. This hypothesis was previously verified experimentally.^26,27^ The application of this concept to a microstructured reversible interface led to the fabrication of arrays of adhesive and disjoining domains when the cell interacts with many such domains. This concept received further elaboration in this work. Considering the cell dimensions, the size of the domains should be about 5 μm or below, as illustrated in Figure 3, with two representative cases of spherical cells and extended cells on the micropatterned PNIPAM brush.

Patterned brushes were previously fabricated using photo- and e-beam lithography, soft lithography, colloidal lithography, and different variations of the scanning probe microscopy (SPM) methods.^32−34^ Photo- and colloidal lithography are scalable methods. Particle aggregation, polydispersity of particles, and random deposition of particles limit colloidal lithography accuracy. Photolithography (typically UV-lithography) provides regular structures and flexibility for selecting pattern shapes and dimensions. In this work, we selected photolithography as one of the most scalable technologies for micromanufacturing. The major drawback of photolithography is a resolution limit of about 0.5 μm.

### Fabrication of Stimuli-Responsive Patterned Brushes

Figure 2 provides the schematic for fabricating the micropatterned PNIPAM brush grafted between the adhesive photoresist SU-8 pillars. The surface of the Si-wafers was functionalized with the ATRP initiator and then coated by a 0–100 nm film of the SU-8 photoresist. The UV irradiation through the photomask with the following development of the film led to the micropatterned surface with SU-8 domains. The ATRP initiator was used to grow PNIPAM brushes on the unexposed areas of the Si-wafers. The SU-8 pillars were functionalized with PEI to generate positively charged adhesive domains of the reversible interface.

### Characteristic Dimensions of the Patterned Brushes

**Figure 4:**
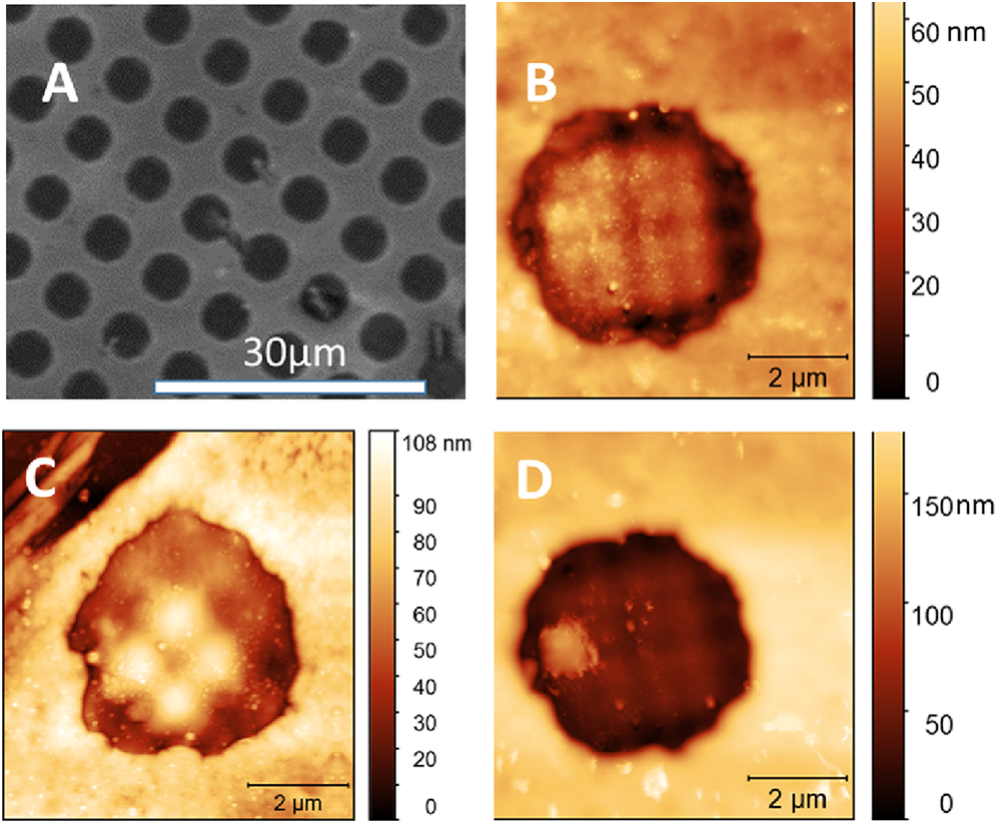
SPM images of microstructured polymer films on a silicon substrate. Domains–epoxy-based photoresist (SU-8), matrix–grafted PNIPAM, dark line on the side–scratch made by a razor blade to obtain the zero level (silicon substrate); (A) SEM image of the surface in vacuo at 25 °C, (B) PNIPAM-SU-8 microstructured surface in air at 25 °C, (C)– PNIPAM-SU-8 microstructured surface in water at 40 °C (above LCST), and (D) PNIPAM-SU-8 microstructured surface in water at 25 °C (below LCST).

**Figure 5:**
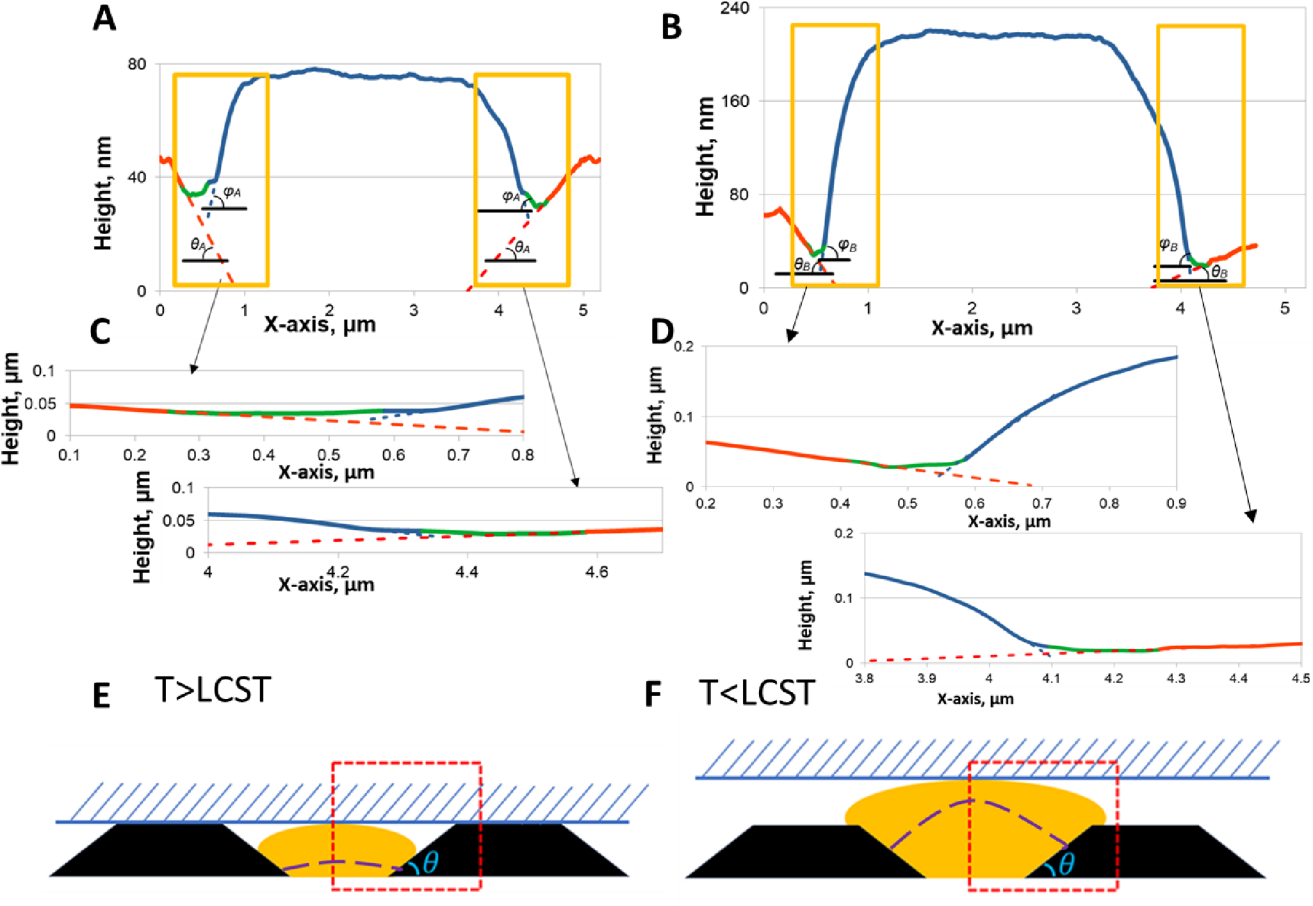
Cross-sectional profiles of the reversible interface present the PNIPAM brush domains between two SU-8 domains obtained with SPM in air (A, C) and in water at 25 °C (B,D) presented in the traditional SPM *Z* and *X* axes scales (A,B) and in the same scale of *Z* and *X* axes (C, D). Schematic of the contact geometry of the flat particles at the interface (E, F). SU-8 pillars are displayed in black and PNIPAM brush in orange regions the bottom surface of a particle is shown as a blue dashed line. The boundaries of the simulation box containing the boundary between the brush and pillars is sketched via red dotted rectangles. The particle is adsorbed on the pillars (*T* > LCST) (E); it is desorbed after swelling of the PNIPAM brush (*T* < LCST) (F). The orange region presents a case when the swollen brush extends over the SU-8 pillars. The pink dash line shows a case when the swollen brush does not extend over the pillars but swells to a height sufficient to push particles off the pillar.

We fabricated patterned brushes with different *h*/*H* ratios (Table 1), where *h* is the thickness of the PNIPAM brush in the dry state and *H* is the thickness of the SU-8 structures and different lateral dimensions of the adhesive SU-8 and disjoining PNIPAM domains. We applied the photomasks with a square shape of the transparent domains 3.5 × 3.5 μm^2^, 4 × 4 μm^2^, and 5 × 5 μm^2^ with spacing between them in a range of 2.5–3.5 μm masked by the dark areas. The dimensions were estimated using SPM topographical images obtained in air and water at 25 °C (*T* < LCST) and at 37 °C (*T* > LCST) (Figures 4 and 5). The characteristic dimensions of the periodic structures, including *h*, *H*, the width of the SU-8 square pillar *D*, and the space between the pillars are shown in Table 1.

Figures 4 and 5 demonstrate the structure and crossectional profiles of the reversible interface when the PNIPAM is in the collapsed and swollen (*T* < LCST) states. The profiles in Figure 5A,B present the commonly used SPM software approach when the *Z*-coordinate is on the nanometer scale and the *X*-coordinate is on the micrometer scale (at a larger scale see Figure S3). The profiles plotted at the same scale of the *Z* and *X* coordinates are shown in Figure 5C, D, which provide a better visualization of the structure that clearly shows the substantial deviation of SU-8 pillar geometry from the geometry of a vertical pillar. This deviation is a result of the optical photolithography resolution limit (500 nm or greater). From the SPM profiles, we estimated the angles formed by SU-8 (θ) and PNIPAM (φ) in the point of their contact as shown in Figure 5C,D. For such low θ and φ angles of 2–5°, the PNIPAM brush located between the SU-8 structures can be considered as a brush patch grafted to an almost flat surface. In such a structure, the edges of the brush splay and form a parabolic profile (Figure 5C,D).

It was demonstrated experimentally^35^ and with molecular dynamic simulations^36^ that the brush thickness in such patches increases with the footprint size and approaches the thickness of the bulk brush if the print size is greater than 1–2 μm, depending on the brush characteristics. For the PNIPAM structures synthesized for this work, the period is 7–8 μm with the width of interpillar domains 2.5–3.5 μm. Consequently, the brush approaches the greatest thickness in the middle of the brush domains (Figure 5A,B). Schematically, the structure of the reversible interface at temperatures below and above the LCST is shown in Figure 5E,F.

Swelling of the microstructured PNIPAM brush when the temperature falls below LCST can be characterized by changes in the brush thickness in the middle of the brush domains (Table 1) and by changes in the footprint area of the SU-8 domains (not covered by the PNIPAM brush) (Table S2). The data were obtained from analysis of the SPM images. The decrease in the open SU-8 surface area depends on the *h*/*H* ratio and varies from 5 to 40% when the temperature drops below LCST. The swollen PNIPAM brush expands over the SU-8 structures due to the low inclination angle of the edges of the SU-8 domains. The angle formed by the brush edge substantially increases at *T* < LCST.

The data of Table 1 can be used to verify if the samples match the discussed above geometrical condition for the reversible interface: 1/*R*_B_ < *h*/*H* < 1/*R*_A_. These conditions are applied to disk-shaped particles (e.g., extended cells on the surface). All the samples except S2 satisfy the condition *h*/*H* < 1/*R*_A_. In the latter case, the PNIPAM brush is too long, preventing the particle or cell from binding to the SU-8 domains at *T* > LCST. All the samples satisfy the condition 1/*R*_B_ < *h*/*H*.

For spherical particles, we should take into account the particle radius and the structure’s lateral dimensions. In the latter case, the condition for the reversible interface can be written as *G*_B_ < *h*/*H* < *G*_A_, where *G*_A_ and *G*_B_ are the structural parameters (Table 1 and Figure S4). All the synthesized samples fall in the range of structural characteristics that match the conditions for the reversible binding of spherical particles except S6 with a too-short PNIPAM brush that cannot swell enough to push off the particles of 37 μm radius used in the experiments discussed below. Importantly, the above-mentioned conditions are based on the geometry of the interacting materials and do not consider the balance of adhesive and disjoining forces. These conditions define the structural characteristics required for the reversible interface, but other critical characteristics are the ratio between the adhesive and disjoining force and the elastic properties of the particles interacting with the reversible interface. If the adhesive force depends on the contact area and interactions in the contact areas, the disjoining force is defined by both the PNIPAM brush molecular characteristics and the disjoining area. This analysis is not the focus of this work.

Interestingly, for soft particles that change their structure after binding to the surface, the particle binds to the surface in a spherical shape but detaches in a disk-like shape. The conditions for the reversible interface should be a combination of the requirements for the disk-shaped and spherical particles: 1/*R*_B_ < *h*/*H* < *G*_A_. This statement has not yet been verified in this work.

### Computer Simulations of the Reversible Interface

According to the experimental analysis of the structure of the reversible interfaces (Figure 5), the SU-8 fragments have the shape of pillars with sloped sides. A particle adsorbs on top of each pillar at *T* > LCST. Upon swelling of the PNIPAM chains at *T* < LCST, a brush not only extends in the vertical direction but also spreads over adjacent sides of the SU-8 pillars. The length scale of this spread is restricted by *R*_N_, the end-to-end distance of individual chains in their swollen state. This effect impacts the vertical component of the force exerted by a brush on the particle, which is the principal mechanism for their desorption from the SU-8 pillars at *T* < LCST. The magnitude of this impact will depend on the very details of the mixed patterned surface geometry. The in-scale modeling of the patterned surface and particle is prohibitively expensive, even in terms of the coarse-grained particle simulations, as explained in detail above. Therefore, in this study, we concentrate on the most critical region, namely, the brush-SU-8 boundary, and examine the role of the pillar angle, θ, on the desorption process, as sketched in Figure 5E,F. These schematics show two representative cases (1) when the brush swells and extends over some fraction of the pillar’s top and (2) when the brush swells and extends over some fraction of the sloped side of the pillar; however, the middle of the swollen brush between two pillars elevates above the pillar’s height (dash line). The third representative case (not shown in Figure 5) is when the swollen brush does not reach the level of the pillar’s height.

In the *first stage* of simulations, we consider the brush, water, and adjacent side of an epoxy pillar with its slope given by the angle θ. Two extreme cases, θ = 5° and θ = 80° are shown in Figure S5A, B in the collapsed state of a brush, at *T* = 37 °C. The height of a flat part of a brush profile is found to be practically independent of the angle θ, which must be attributed to the fact that *R*_N_ ≪ *d* in a collapsed state of chains. Therefore, the same estimate for the average brush height, *h*_*s*_ ≈ 16 in a collapsed state, obtained from these results, can be used at all angles θ, as shown via a red dotted line. To validate sufficient dimension, *L*_Z_, of the simulation box, the same simulations are undertaken in a swollen state at *T =* 25 °C (Figure S5C,D), for the same cases of *θ =* 5° and *θ =* 80°. In both cases, the brush profile does not reach the top of the box; therefore, the simulation box height, *L*_*Z*_*=* 40 is reasonable.

In the *second stage* of the simulations, we cut the SU-8 pillar at *z* = *H* and introduced a membrane that mimics the bottom surface of the adsorbed particle. Both soft and rigid membranes comprise a regular grid of 4422 beads arranged in the sites of the square lattice, with the separation between the nearest neighbors equal to *s =* 0.49. All nearest neighbors of a membrane are bonded via force (eq 3) with the fixed bond distance *b*_ij_ and a bonding strength of *k*_B_*=* 200. In contrast to the polymer beads, bonded beads of the membrane do not repulse each other via force (eq 1) to ensure that the equilibrium bond length is strictly equal to *s*, to avoid self-bending of a membrane. For the case of a rigid membrane, an additional bending force (eq 4) is applied with the bending constant set equal to *k*_a_*=* 10^4^ and θ_0_*=* 0. The setup is schematically shown in Figure 5E,F, where we choose the pillar height *H* equal to the brush height, *h*_s_ = 16, in its collapsed state.

The results obtained for the simulation setup with the membrane for *T* = 37 °C are shown in Figure S6. The snapshots confirm that for the case of H = *h*_s_ = 16, the collapsed PNIPAM brush is indeed completely contained within the available region of a simulation box that is restricted by both the SU-8 pillar and the membrane at both angles θ = 5° and 80°.

In the third stage of the simulations, we decrease the temperature from *T* = 37 °C down to *T* = 25 °C. This was implemented via a change in the polymer–water repulsion strength *a*_ij_ (eq 1) from *a*_ij_*=* 38 down to *a*_ij_*=* 25.6, which resulted in the hydration of PNIPAM and swelling of the brush. The membrane profile, which is initially at the top of a polymer brush, develops as a result of the forces generated by the swollen brush.

**Figure 6:**
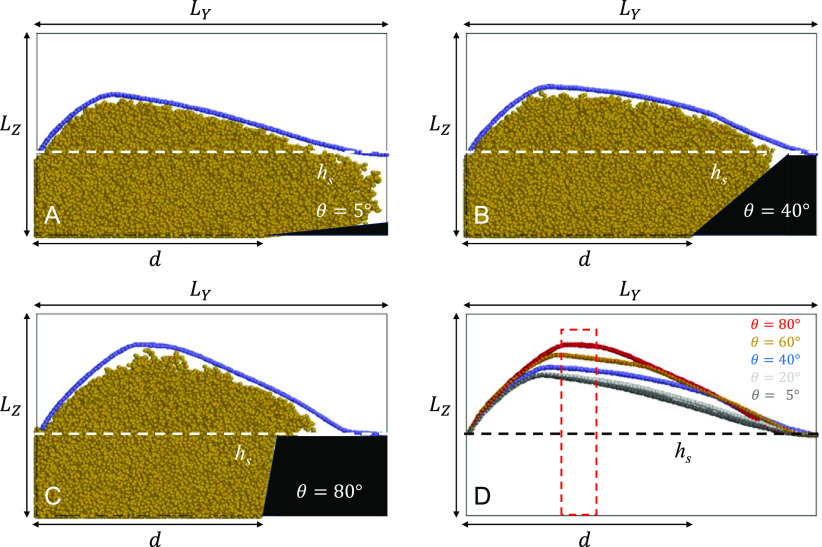
Profiles for the case of a soft pinned membrane swollen at *T* = 25 °C, θ = 5° (A), 40° (B), and 80° (C) after 8 × 10^5^ DPD steps. (D) Color-coded membrane profiles. The white or black dashed line shows the brush height, *h*_s_, at *T* = 37 °C. The red dashed rectangle shows the pseudobulk region of the brush distanced from both pinned sides.

We considered the soft membrane first. In our simulations, it is pinned by both edges adjacent to the *y* = 0 and *y = L*_*Y*_ walls by setting the mass of the beads adjacent to these edges equal to *m* = 10^7^, 2 orders of magnitude higher than that for the rest of the membrane beads. The results of the simulations at various angles θ are shown in Figure 6 frames. The red dashed rectangle in snapshot D shows the brush area distanced from both pinned sides, which can be interpreted as a pseudobulk region of the brush.

The membrane profile bends strongly in the middle of the simulation box while keeping the positions of its left and right edges fixed due to pinning. The exact shape of a profile is the result of the competition between the force acting from the brush and the membrane’s elasticity. For a small θ, the mass of a brush spreads further over the pillar side on the right side of the simulation box. This reduces the force component along the *OZ* axis in the region adjacent to the pillar, resulting in a decrease in the membrane profile there. The height of the membrane profile in the pseudobulk region (Figure 6D, red dashed rectangle) is also higher for larger values of θ. We do not know whether this is a consequence of the simulation box not being long enough along the *OY* axis. In any case, the simulations show a visible dependence of the soft membrane profile on the pillar angle θ.

**Figure 7:**
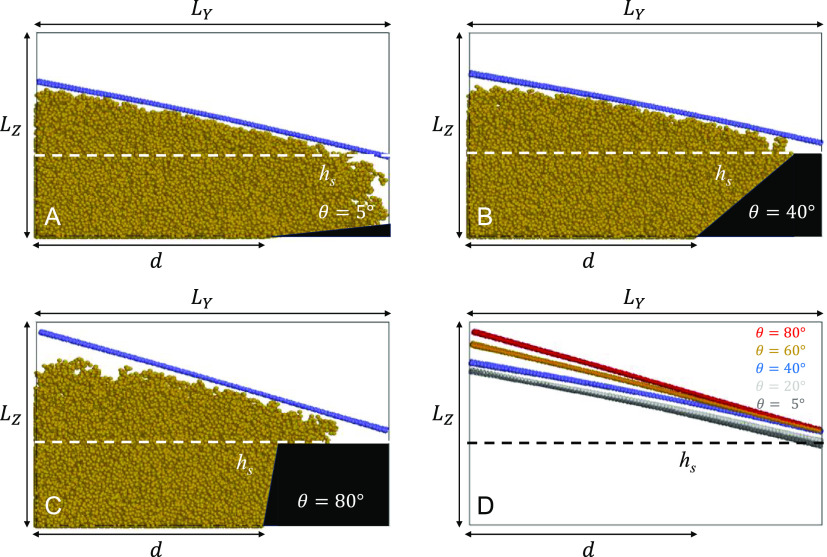
Profiles for the case of a rigid unpinned membrane swollen at *T* = 25 °C, θ *=* 5° (A), 40° (B), and 80° (C) after 8 × 10^5^ DPD steps. (D) shows the color-coded membrane profiles. The white or black dashed line shows the brush height, *h*_s_, at *T* = 37 °C.

For a rigid membrane, the edges are set free, allowing the movement of a membrane as a single unit. It mimics the bottom surface of a particle, and its rigidity is ensured by engaging with the bending force (eq 4). The results of the simulations at various angles θ are shown in Figure 7.

Similarly to the case of a soft membrane, we also see the dependence of the overall profile height of a rigid membrane on angle θ. The swelling stages of the brush and changes in the membrane position with time are shown in Figures S7 and S8. The membrane tends to form an angle with respect to the *OY* axis. This is attributed to both the finite dimensions of the simulation box and the membrane rigidity. For the box with a larger value of *L*_*Y*_, and involving a larger SU-8 pillar, such an angle would not be observed. Instead, the membrane is expected to be lifted parallel to the *OY* axis, but this simulation setup is reserved for a future study.

The initial state was obtained at *T* = 37 °C. Then the temperature was lowered to *T* = 25 °C. At the beginning of swelling, the brush redistributes its mass toward the side of the SU-8 pillar. This costs less energy than lifting the membrane up. The difference between θ = 5° and θ = 80° is that in the first case, the brush does not reach the top surface of the pillar and spreads over its side surface (Figure S7). In the second case, the brush first extends to the top surface of the membrane (Figure S8) and then lifts the membrane up.

**Figure 8:**
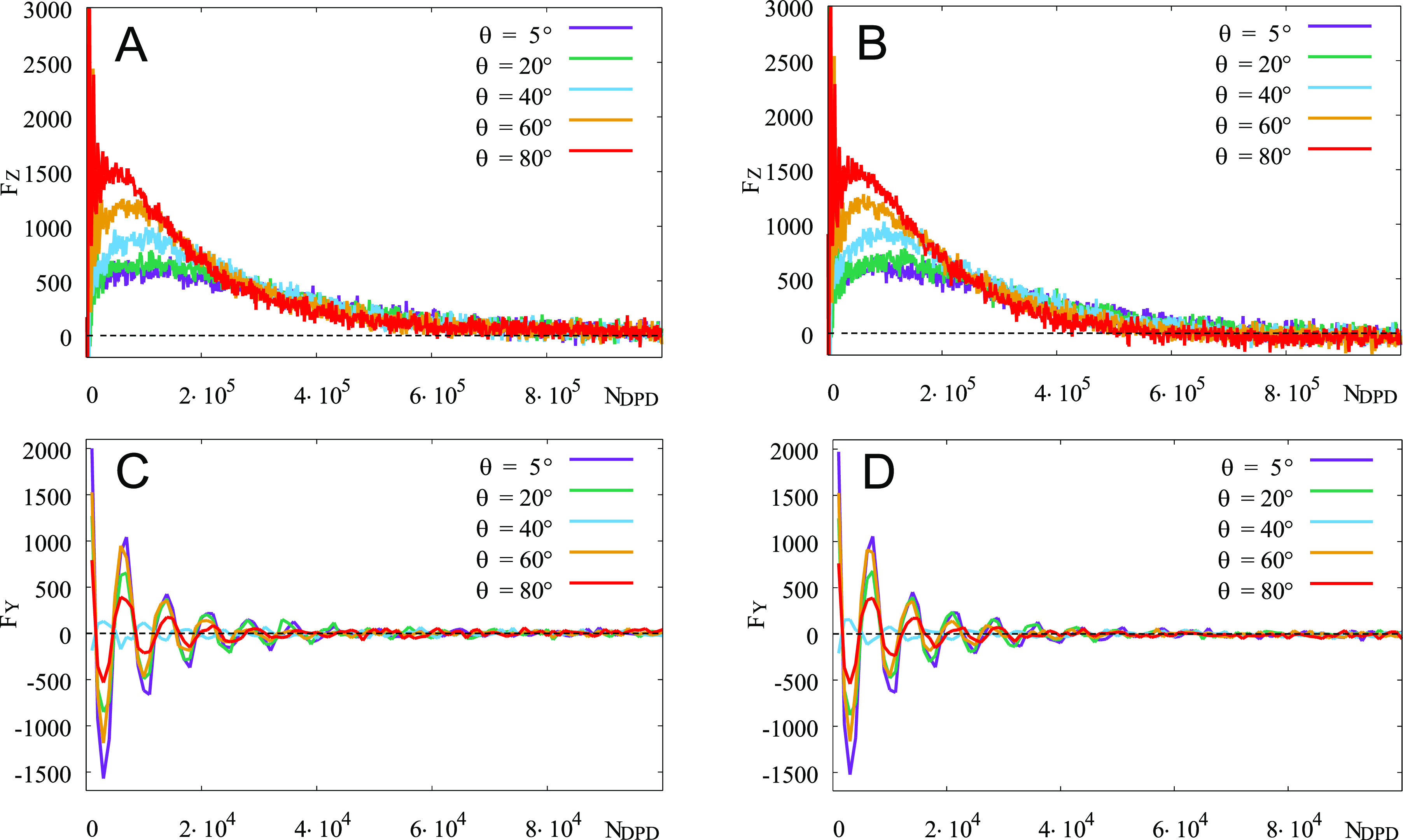
Time evolution of the *OZ* (A,B) and *OY* (C,D) components of the force acting on the soft (A,C) and rigid (B, D) membranes by the swollen polymer brush upon its relaxation at *T* = 25 °C.

The force generated by the swollen brush results in a certain amount of deformation and then lifting of the membrane up. The plots in Figure 8 demonstrate the development of *F*_*z*_ and *F*_*Y*_ – the *OZ* and *OY* components of the forces for the soft and rigid membrane.

Both cases of a soft and rigid membrane provide practically indistinguishable results for the force. The *F*_*z*_ component demonstrates an initial spike and a local maximum after about 10^5^ DPD steps, with the following decay to zero after 8 × 10^5^ DPD steps. Its maximum is found at approximately 10^5^ DPD steps for the SU-8 domain slope of θ = 5°, and shifts to an earlier instance of about 5 × 10^4^ DPD steps for the steeper slope of θ = 80°. At the same time, the value of *F*_*z*_ at its maximum almost triples for the latter case. *F*_*Y*_ component shows an interesting oscillatory behavior, which occurs at the early stage of swelling (up to 5 × 10^4^ DPD steps). The amplitudes of these oscillations decrease with the increase of θ when 5° < θ < 35°, and demonstrate an opposite behavior for 35° < θ < 80°. The explanation of the *F*_*z*_ and *F*_*Y*_ behavior is based on the presence of two stages of brush swelling, as discussed above. The *first stage* is associated with moving the brush monomers along the *OY* axis, resulting in covering the slope of the SU-8 pillar. This imposes a drag force, *F*_*Y*_, on the membrane beads and initiates their oscillations. This stage is possible for moderately steep sides only, θ < *35*°, and the amplitude of *F*_*Y*_ oscillations decrease with the increase of θ until it reaches 35°. With a further increase of θ, the space available for a brush diminishes: a brush starts to swell along *OZ* axis earlier, and the force is distributed mainly upward, comprising the *second stage* of swelling. This explains both the shift of the *F*_*z*_ maximum toward earlier times and the growth of its maximum with the increase of θ. At these conditions, one also observes some increase in the amplitude of the *F*_*Y*_ oscillations. Therefore, the force dynamics are in accord with the time evolution for swelling of the brush confined by the membrane (Figures S7 and S8).

### Experimental Study of Reversible Binding of Solid Particles

The properties of the reversible interfaces were experimentally tested by the estimation of the shear stress to detach surface-bound PS beads. The PS beads of 37 μm in the radius are decorated with sulfo-groups, and hence, they carry a negative charge in aqueous solutions. The surface of the SU-8 domains is decorated with PEI, which carries a positive charge. We adjusted the pH and experimentally found that at pH 9 the electrostatic interaction between the beads and the SU-8 domains is minimized to the level that can be measured by variation of the liquid flow in the fluid cell used for this study. The density of the PS beads is slightly above 1 g × cm^–3^. The particles precipitated and bind to the sample surface in the aqueous dispersion. After 3 min of incubation time, the liquid flux in the cell was increased stepwise after a 3 min delay after each increase of the flux. Every 3 min, an optical image of the PS beads on the surface was recorded and analyzed to quantify the number of particles and surface coverage.

**Figure 9:**
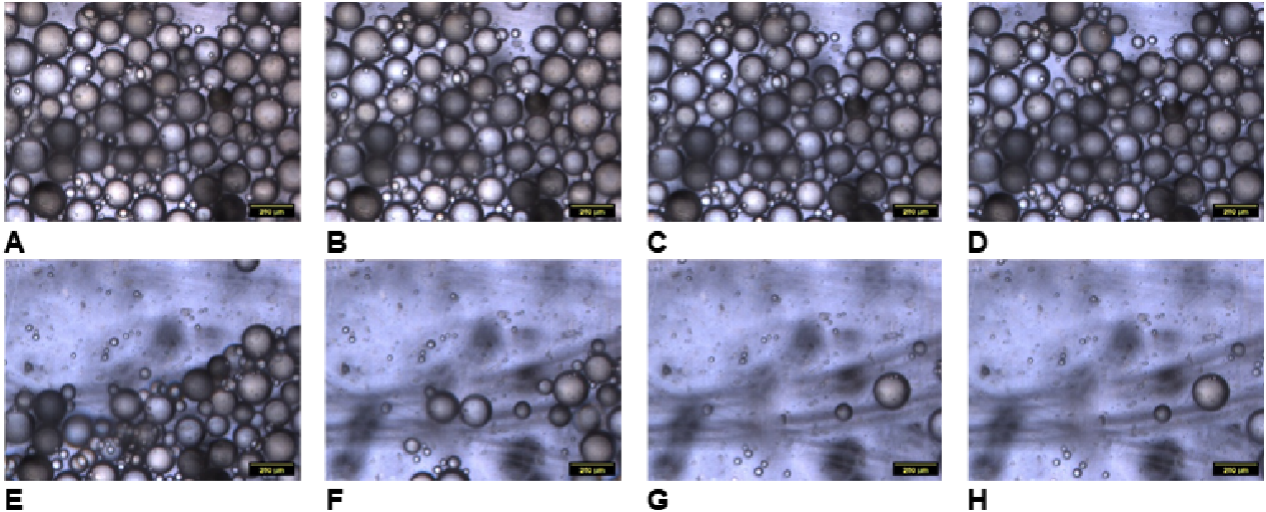
Optical images of the PS beads on the reversible interface sample S9 (*h*/*H* = 1.24), 37 °C: A, B, C, D, E, F, G, and H, at different wall shears of 0.45, 0.93, 1.42, 1.90, 2.39, 2.87, 3.36, and 3.84 Pa, respectively. The scale bars are 200 μm.

**Figure 10:**
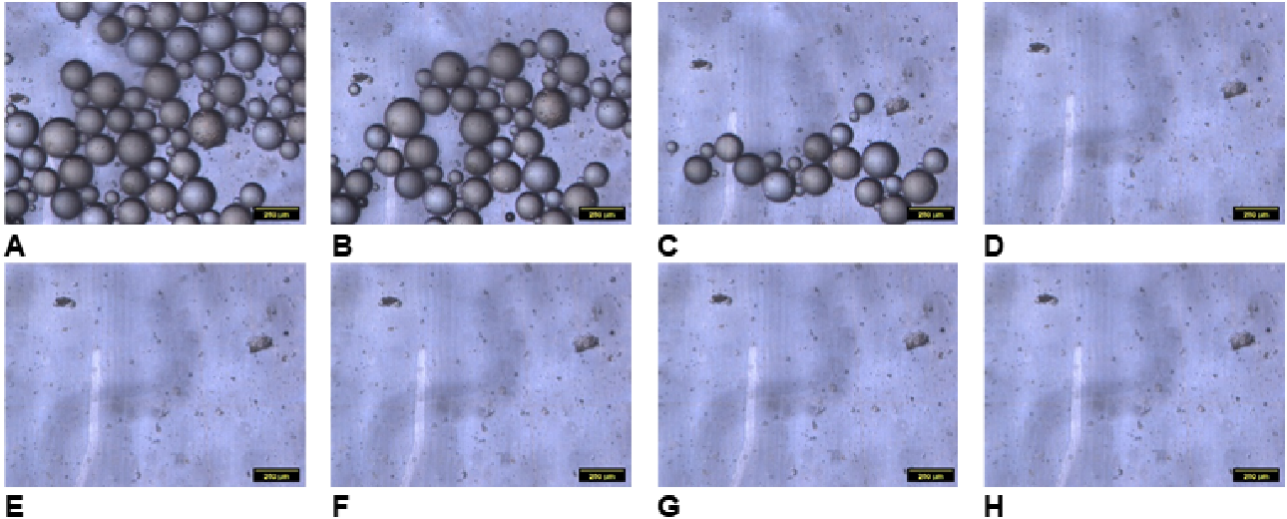
Optical images of the PS beads on the reversible interface sample S9 (*h*/*H* = 1.24), 25 °C: A, B, C, D, E, F, G, and H, at different wall shear stresses 0.65, 1.35, 2.05, 2.75, 3.45, 4.15, 4.85, and 5.55 Pa, respectively. The scale bars are 200 μm.

We used three samples for these experiments, S5, S7, and S9, with different *h*/*H* ratios of 0.53, 1.13, and 1.24, respectively. All the samples satisfy the condition *G*_B_ < *h*/*H* < *G*_A_. Additionally, we studied samples S2 and S6 that do not satisfy this condition and control surfaces with only SU-8_PEI and only the PNIPAM brush, serving as references. The experiments were conducted at 37 and 25 °C. The representative series of the images collected from the experiment with S7 is shown in Figures 9 and 10 at temperatures above and below LCST, respectively. The results obtained for other samples and controlled surfaces can be found in the SI (Figures S9–S21).

**Figure 11:**
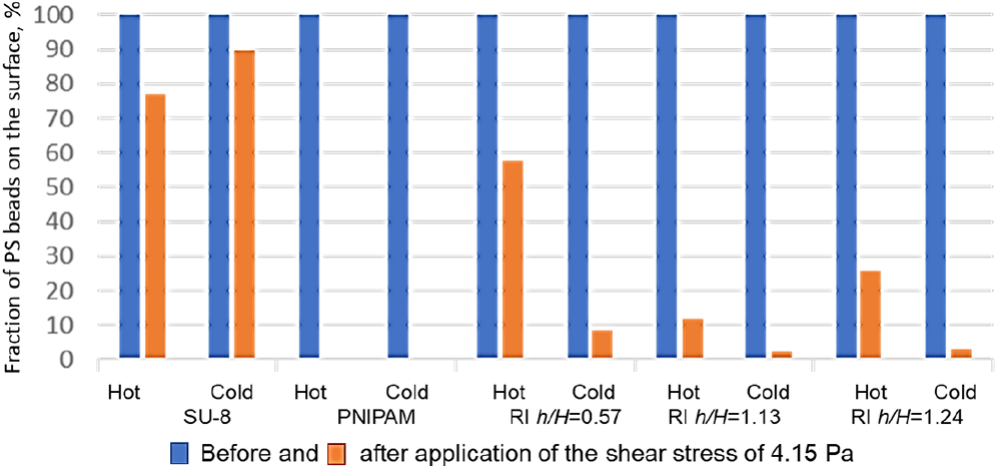
Fraction of the PS beads that are retained on the surface after application of a 4.15 Pa shear stress.

The results for all of the experiments are summarized in Table 2 and Figures 11, S23 and S24). We present the shear stress to detach 50% of particles from the surface (Table 2) and the fraction of the particles retained after the application of 4.15 Pa shear stress (Figure 11). Only samples that satisfy the reversible adhesion conditions are analyzed in Figure 11. The information for all ranges of shear stress can be found in SI.

**Table 2:** Shear Stress to Detach 50% PS Beads From the Reversible and Control Interfaces (Standard Deviations are Provided in Brackets)

		shear stress, Pa	
sample	*h*/*H*	*T* = 37 °C	*T* = 20 °C
SU-8, control	-	>5	>5
S2	2.18 (0.23)	2.1	1.2
S5	0.57 (0.2)	3.1 (0.25)	2.8 (0.15)
S6	0.24 (0.05)	>5	>5
S7	1.13 (0.08)	2.2 (0.3)	1.8 (0.2)
S9	1.24 (0.14)	2.6 (0.2)	1.7 (0.15)
PNIPAM, control	-	0.9 (0.2)	1.25 (0.2)

The premise of the test is that the higher the PNIPAM to SU-8 *h*/*H*, the greater the mechanical force that loosens the attachment of the particles to the surface and the lower the flow rate required to dislodge the particles. The SU-8_PEI control sample surface has a high affinity to the PS beads; the beads cannot be detached by the maximum achievable shear force in the experimental setup. The same result was obtained for sample S6 with an *h*/*H* ratio that was too small to push off the particle. The PNIPAM control surface has a low affinity to the PS beads because of the steric repulsion of the PNIPAM brush. The same results were obtained at *T* < LCST for the particle detachment from the PNIPAM reference sample and S2 sample with a high *h*/*H* ratio. The higher adhesion of the particles to S2 surface than that for the PNIPAM brush at *T* > LCST can be explained by, in this case, the *h*/*H* ratio is 2.18, and it is close to *G*_A_ = 1.94. Some nonuniformity of the microstructured surface may result in SU8 areas that can be accessible for the particles. The adhesion of the PS beads to both control surfaces is not affected by temperature. In contrast, the reversible interfaces clearly demonstrate the effect of temperature. The shear stress to detach particles drops 2-fold after the temperature decreases below LCST. At relatively high shear stresses of 4.15 Pa and at *T* < LCST, most particles were removed from all three samples while they remained on the SU-8_PEI control sample (Figure S10) and S6 sample (Figure S18).

The obtained results are consistent with the proposed criteria for the microstructured reversible interface and computer simulations. The disjoining pressure weakened particle-interface interactions over a broad range of *h*/*H* ratios and even at a low slope of the SU-8 domain side wall, providing a certain level of flexibility for fabricating such materials. This effect is stronger for higher *h*/*H* ratios.

## Conclusions

We fabricated and examined the properties of the reversible interface designed by using adhesive PEI functionalized SU-8 domains and thermoresponsive PNIPAM brush disjoining microdomains. We proposed a simple structure-based criterium for reversible adhesion, which is the *h/H* ratio (dry brush thickness/adhesive domain thickness) when temperature is switched from *T* > LCST to *T* < LCST. For disk-shaped particles, 1/*R*_*B*_ < *h*/*H<*1/*R*_A_, and for spherical particles *G*_*B*_ < *h/H < G*_*A*_, where *R*_A_ and *R*_B_ are the swelling characteristics of the brush, and *G*_A_ and *G*_B_ are the structural parameters that include the size of the interacting particle, as discussed in the article. The separation of adhesive and disjoining domains allows for expansion of the range of structural characteristics of the reversible interface. This can be promising for scaling up the thermoresponsive materials for cell harvesting applications where precise structural dimensions are not approachable or affordable because of the resolution limits of simple optical lithography. The developed recommendations can be applied to spherical and disk-shaped colloidal particles and live cells.

The complementary computer simulations provided information that experiments cannot easily achieve. The simulations revealed the PNIPAM brush behavior on the microstructured surfaces with different profiles of SU-8 domains. The results demonstrated that even at a very high deviation of the SU-8 pillars from the ideal vertical wall structure, the swollen PNIPAM brush could occupy all of the volume between the microstructured interface and an adhered particle and develop a disjoining pressure to detach adsorbed particulates. In addition to the vertical disjoining force, a shear force component contributes to particle detachment. The disjoining force is identical for rigid and elastic particles; however, it depends on the slope of the SU-8 domains, as was observed in the simulations and experiments. The shear component can impact the detachment mechanism in the case of direction-sensitive adhesion and, consequently, may improve the detachment efficiency. The simulations demonstrated for elastic particles (soft colloidal particles) that the disjoining pressure is nonuniformly distributed over the contact surface area, which may also affect the detachment mechanism of soft colloids and cells. The latter effect will require a special experimental setup and analysis in future work.

## Supporting Information Available

Experimental setup to study particle reversible adhesion, characterization of PS beads, structural conditions for the reversible interface, and results of particle adhesion tests (PDF)
