# Supplementary material for: Reversible Binding Interfaces Made of Microstructured Polymer Brushes
Source: Langmuir. 2024 Mar 25;40(13):7008–20. doi: 10.1021/acs.langmuir.4c00062 (PMC10993409; doi:10.1021/acs.langmuir.4c00062)
Supplement: Supplementary file 1 — la4c00062_si_001.pdf [file la4c00062_si_001.pdf]

Supporting Information

Reversible Binding Interfaces Made of Microstructured Polymer Brushes

Ronaldo Badenhorst,[1] Sergei Makaev,[1] Dmytro Yaremchuk,[2] Yash Sajjan,[1] Artem Sulimov,[3] Vladimir V. Reukov,[4] Nickolay V. Lavrik[5], Jaroslav Ilnytskyi,[2,6] Sergiy Minko[1]*

[1]Nanostructured Material Lab, University of Georgia, Athens, GA 30602, USA

[2] Institute for Condensed Matter Physics of the National Academy of Sciences of Ukraine, Lviv, 790011, Ukraine

[3]Department of Chemistry, University of Georgia, Athens, GA 30602, USA

[4]Department of Textiles, Merchandising, and Interiors, University of Georgia, Athens, GA 30602, USA

[5]Center for Nanophase Materials Sciences, Oak Ridge National Lab, Oak Ridge, TN 37831, USA

[6]Institute of Applied Mathematics and Fundamental Sciences, Lviv Polytechnic National University, Lviv, UA-79013, Ukraine

*Corresponding Author, Sergiy Minko, Email: sminko@uga.edu

PS Beads Optical Image Analysis

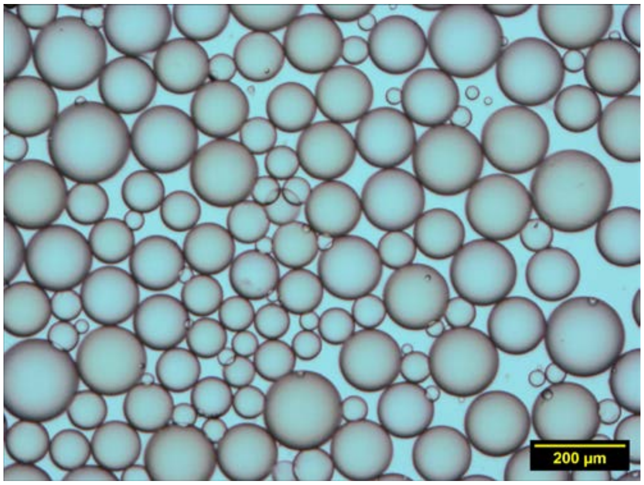

Figure S1. Optical image of the PS beads. The mean radius is 37.4 µm, the median radius is 34.1 µm, the standard deviation is 28.6 µm.

Fluid Cell, Experimental Setup, and Estimation of the Wall Shear Stress

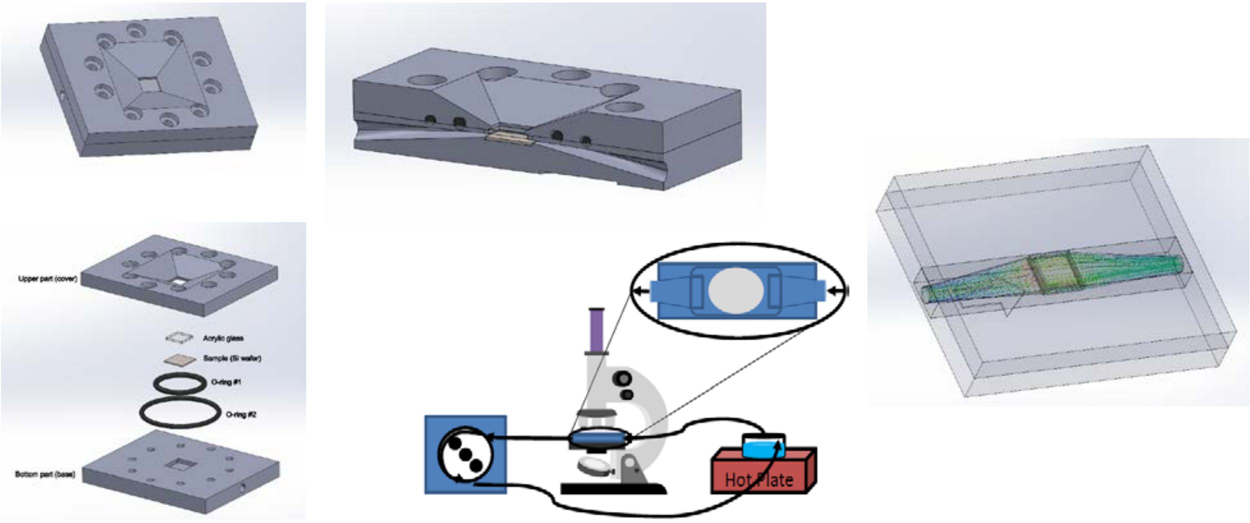

Figure S2. Schematic of the fluid cell and the experimental setup for studying the PS beads adhesion/detachment at the reversible interfaces (the liquid flow channel dimensions: width *w*=10.35 mm, height *l*=0.45 mm).

Table S1. Estimation of the wall shear stress in the fluid cell channel (standard deviations are provided in brackets).

Liquid flow, *Q,* 10^{-7} m^3s^{-1}	Shear stress, 20°C τ*, Pa	Shear stress, 37°C τ*, Pa
2.01 (0.01)	0.58 (0.07)	0.45 (0.07)
4.71 (0.01)	1.35 (0.07)	0.93 (0.07)
7.15 (0.01)	2.05 (0.07)	1.42 (0.07)
9.6 (0.01)	2.75 (0.07)	1.90 (0.07)
12.0 (0.01)	3.45 (0.07)	2.39 (0.07)
14.5 (0.01)	4.15 (0.07)	2.87 (0.07)
17.0 (0.01)	4.78 (0.07)	3.36 (0.07)
19.4 (0.01)	5.55(0.07)	3.84 (0.07)

$*\tau = \frac{6Q\mu}{wl^2}$ (Poiseuille model)**

μ - viscosity of the liquid, μ (20°C) = 0.001 kg $m^{-1}s^{-1}$; μ (37°C) = 0.00069 kg $m^{-1}s^{-1}$;

**Hochmuth, R. M.; Mohandas, N., Blackshear, P.L. Measurement of the Elastic Modulus for Red Cell Membrane Using a Fluid Mechanical Technique *Biophysical J.*, 1978, 13, 747-762.

The shear stress estimate using the analytical equations*** was not more than 5% different from the Poiseuille model.

*** Cornish, R. J. Flow in a Pipe of Rectangular Cross-Section. 1928, *Proc. R. Soc. A* 12o (786): 691-700. ibid GmbH, Application Note 11, https://ibidi-com.webpkgcache.com/doc/-/s/ibidi.com/img/cms/support/AN/AN11_Shear_stress.pdf

The typical wall shear stress to detach cells from the affinity surface ranges from 1 to 400 Pa, while at a low surface concentration of adhesive biomolecules it is in the range of 1-10 Pa (Hang Lu, Lily Y. Koo, Wechung M. Wang, Douglas A. Lauffenburger, Linda G. Griffith and Klavs F. Jensen Microfluidic Shear Devices for Quantitative Analysis of Cell Adhesion, *Anal. Chem.*, 2004, 76(18) 5243-5602).

Sample Characteristics

Table S2. Inclination angles of SU-8 (θ_A, θ_B) and PNIPAM (φ_A, φ_B) structures at the edges of PNIPAM brushes, the open surface area of SU-8 domains (S_A, S_B) above (A in the air) and in the water below(B subscripts) LCST. Standard deviations are given in brackets.

Sample	θ_A°	θ_B°	φ_A°	φ_B°	S_A, µm^2	S_B, µm^2	$(S_A$-$S_B)/S_A$
S1	4.8 (0.62)	5.4 (0.7)	10.1 (2.0)	24.8 (1.3)	11.0 (1.2)	8.8 (1.2)	0.21 (0.07)
S2	1.3 (0.5)	1.2 (0.4)	9.9 (1.4)	30.1 (3.8)	6.7 (1.1)	3.9 (0.9)	0.4 (0.05)
S3	6.3 (1.0)	2.6 (1.0)	5.2 (0.8)	24.1 (1.8)	17.3 (2.0)	13.6 (1.9)	0.22 (0.04)
S5	3.7 (0.6)	3.6 (1.6)	5.0 (0.1)	18.9 (2.9)	22.8 (0.3)	18.3 (0.5)	0.20 (0.01)
S6	7.7 (1.4)	1.9 (0.8)	2.0 (2.1)	6.4 (1.0)	19.6 (0.6)	18.5 (0.7)	0.06 (0.03)
S7	3.4 (0.5)	3.7 (1.5)	4.8 (2.9)	18.8 (2.3)	20.4 (2.1)	17.0 (2.4)	0.17 (0.04)
S8	4.7 (1.9)	2.1 (2.0)	6.4 (2.6)	29.7 (2.3)	17.2 (1.2)	14,3 (1.2)	0.17 (0.03)
S9	2.2 (0.6)	1.4 (0.5)	5.1 (1.1)	17.6 (1.5)	14.5 (1.1)	11.9 (1.0)	0.17 (0.05)

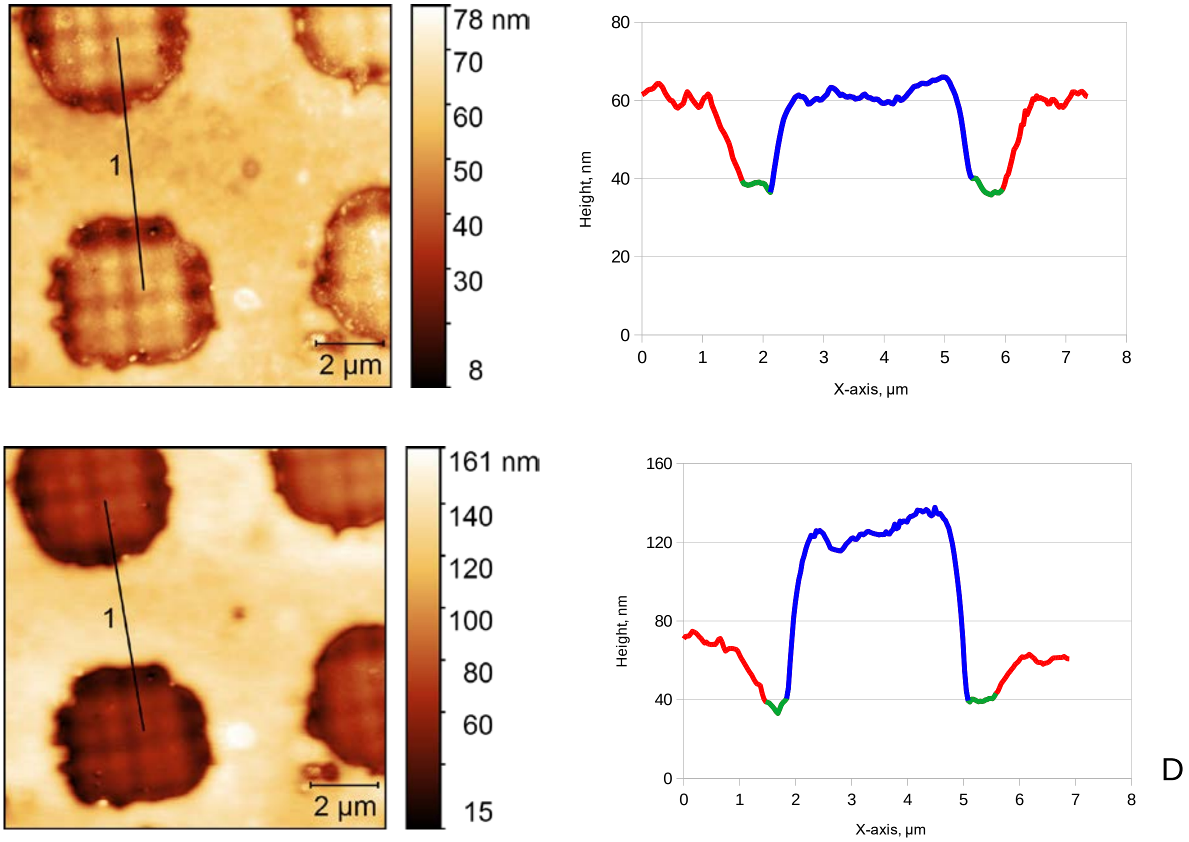

Figure S3. Cross-sectional profiles of the reversible interface present the PNIPAM brush domains between two SU-8 domains obtained with SPM in air (A, B) and in water at 25 ℃ (C,D). Line 1 on SPM images shows the location of the cross-sectional profile. Note 5-10 nm height variation over 5-10 µm range.

Contact Geometry of Spherical Particles on the Reversible Interface.

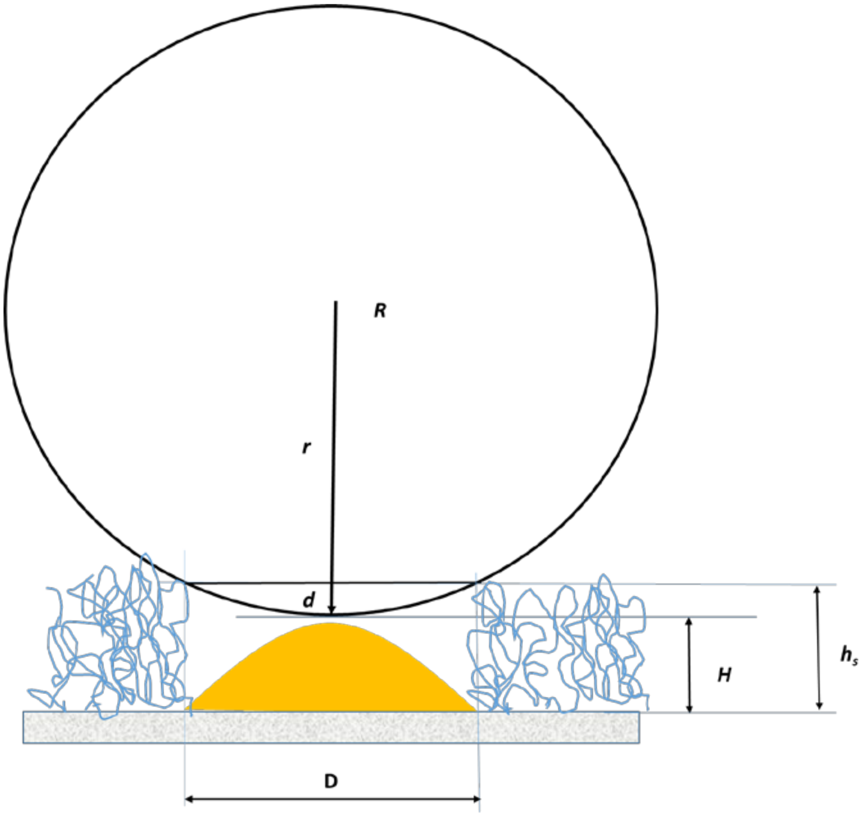

Figure S4. Schematic of the spherical particle bound to the reversible interface

H is the height of the SU-8 domains

h is the height of the dry PNIPAM brush

h_s is the height of the PNIPAM brush in water at T>LCST

h_{sw} is the height of the PNIPAM brush in water at T>LCST

R- is the radius of the particle (PS bead)

D is the lateral size of the SU-8 domain which is equal to the chord of the circle segment

r and d are the height and apothem of the circle segment

Solving the equations

$R = r + d$

$h_s = H + d$

$r = \frac{1}{2}\sqrt{4R^2 - D^2}$ (circular segment geometry)

we obtain:

$h_s = H + R - \frac{1}{2}\sqrt{4R^2 - D^2}$.

Defining the swelling ratios as:

$$R_A = \frac{h_s}{h}$$

$$R_B = \frac{h_{sw}}{h}$$

and applying the conditions

a) for the adhesive contact above LCST
$h_s < H + d$

and

b) for the detachment below LCST

$h_{sw} > H + d$,

we obtain the geometrical conditions for the reversible interface

$$\frac{H+R-\frac{1}{2}\sqrt{4R^2-D^2}}{HR_B} < \frac{h}{H} < \frac{H+R-\frac{1}{2}\sqrt{4R^2-D^2}}{HR_A} \ ,$$

or $G_B < \frac{h}{H} < G_A$

where G_A and G_B are geometric characteristics of the reversible interface above and below LCST, respectively.

if *R*>>*D* (limit is a plate-like particle), the conditions convert into:

$$\frac{1}{R_B} < \frac{h}{H} < \frac{1}{R_A}$$

This Estimation does not consider the contribution of the brush edges for non-vertical walls of the SU-8 structures. The latter can result in an increase of *D*.

Results of DPD Simulations

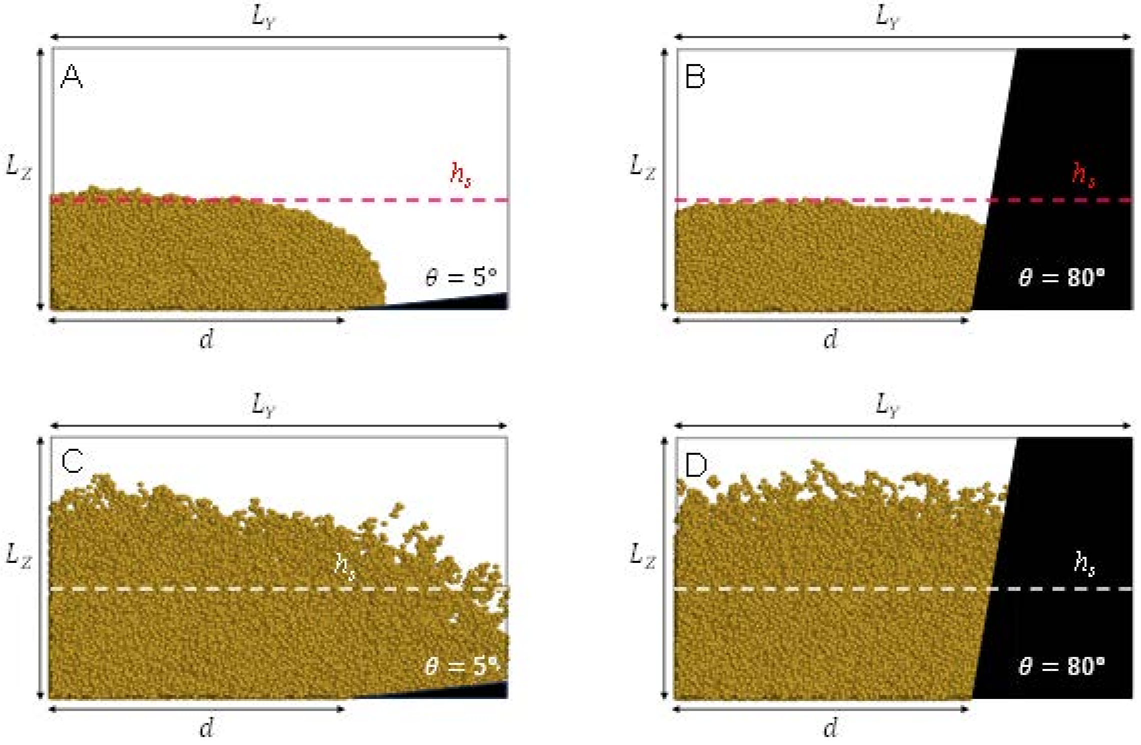

Figure S5. Collapsed (A, B) and swollen (C, D) PNIPAM brush at 37℃ and 25℃, respectively; SU-8 angle $\theta = 5°$(A, C) and $\theta = 80°$(B, D). Red dashed line in both cases: the estimate for the average brush height, $h_s \approx 16$; water is not shown.

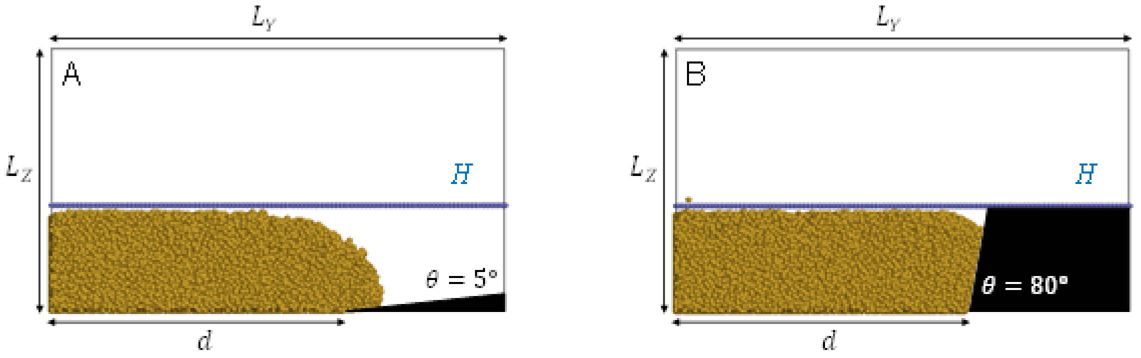

Figure S6. Simulation snapshots showing the profile of collapsed PNIPAM brush at $T = 37$℃ and angles $\theta = 5°$ (A) and $80°$ (B); water is not shown.

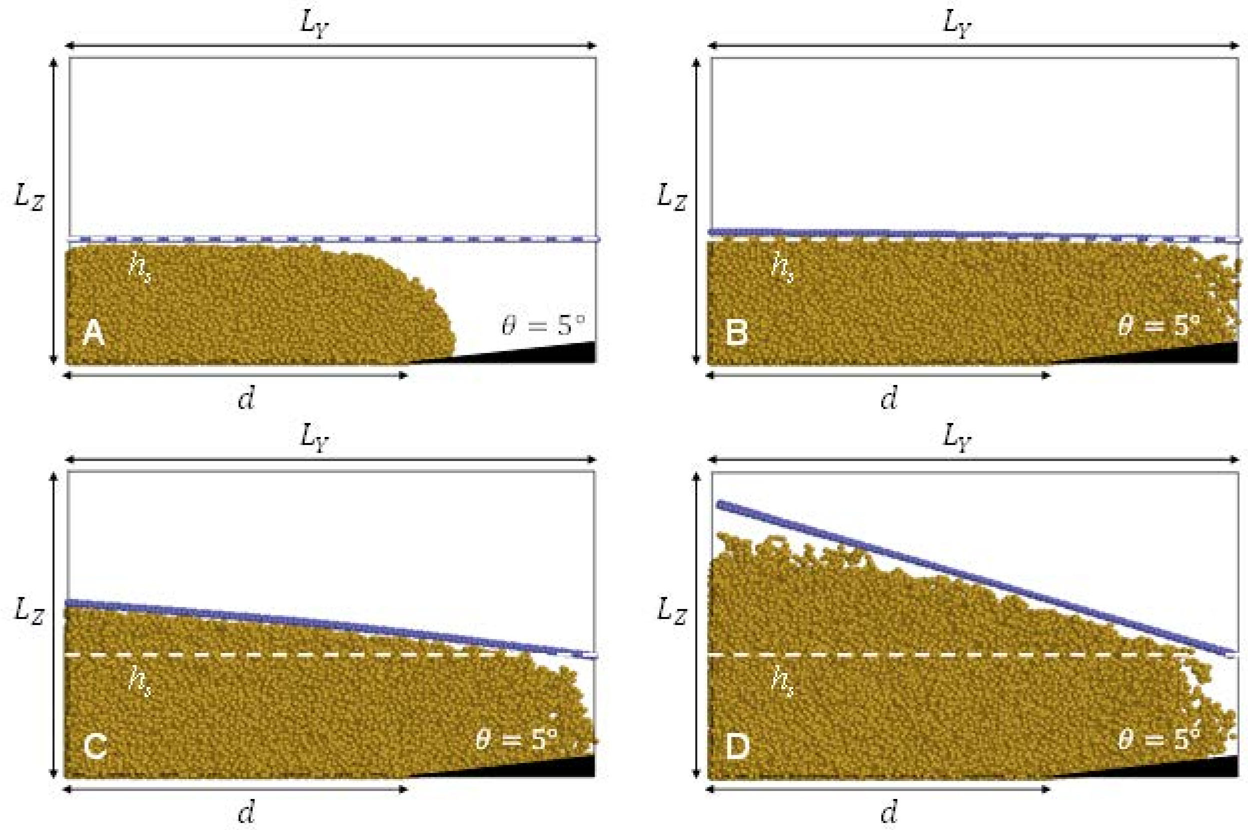

Figure S7. Stages of swelling of the PNIPAM brush at $T = 25$℃ at a small SU-8 pillar slope angle $\theta = 5°$. Initial state (A), after $2 \cdot 10^5$ DPD steps (B), after $5 \cdot 10^5$ DPD steps (C), after $8 \cdot 10^5$ DPD steps (D).

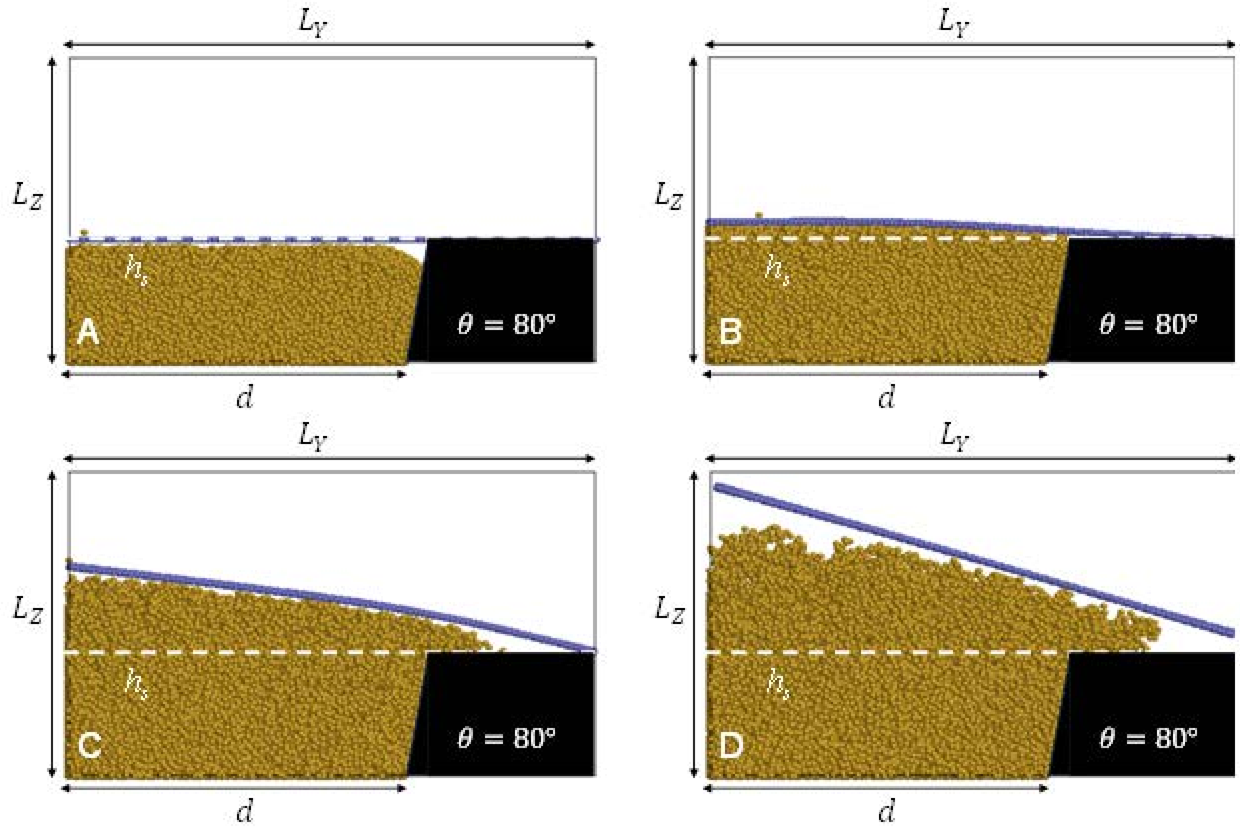

Figure S8. Stages of swelling of the PNIPAM brush at $T = 25$℃ at a large SU-8 pillar slope angle $\theta = 80°$. Initial state (A), after $2 \cdot 10^5$ DPD steps (B), after 5$\cdot$ 10^5 DPD steps (C), after $8 \cdot 10^5$ DPD steps (D).

Experiments on Reversible Adhesion of PS Beads.

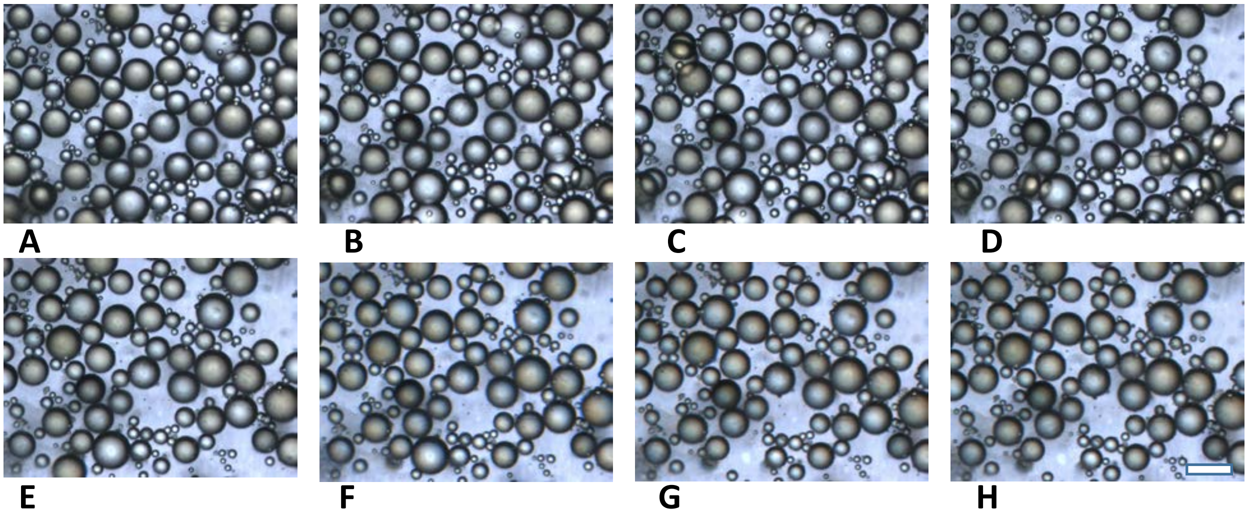

Figure S9. Control experiment. Optical images of the PS beads on the SU-8 surface (37 ^oC) A, B, C, D, E, F, G, H, at different wall shear stresses 0.45; 0.93; 1.42; 1.90; 2.39; 2.87; 3.36; 3.84 Pa, respectively. The scale bar is 200 µm.

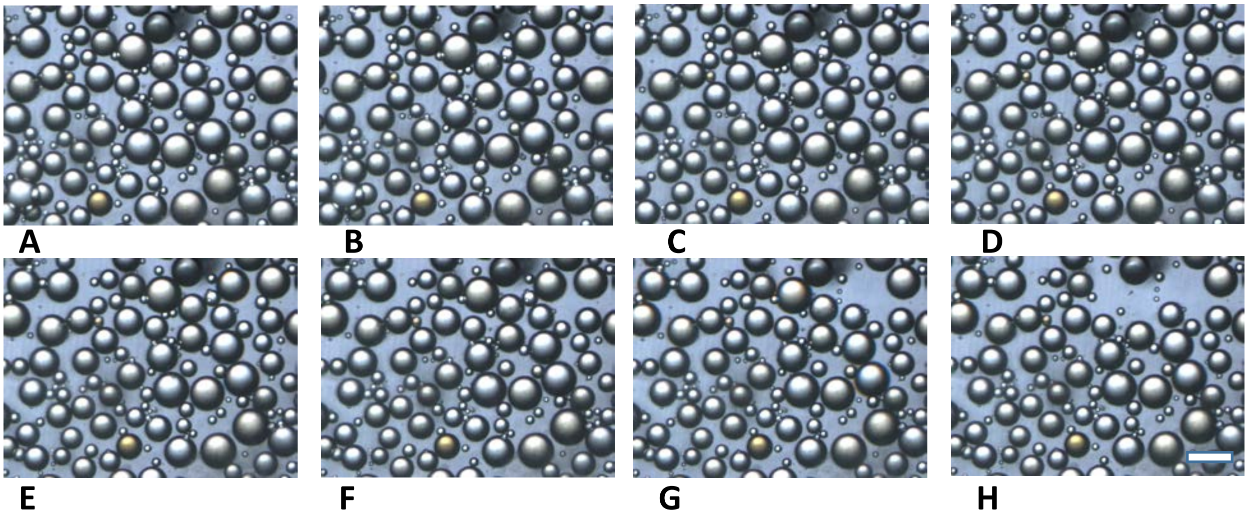

Figure S10. Control experiment. Optical images of the PS beads on the SU-8 surface (25 ^oC) A, B, C, D, E, F, G, H, at different wall shear stresses 0.65; 1.35; 2.05; 2.75; 3.45; 4.15; 4.85; 5.55 Pa, respectively. The scale bar is 200 µm.

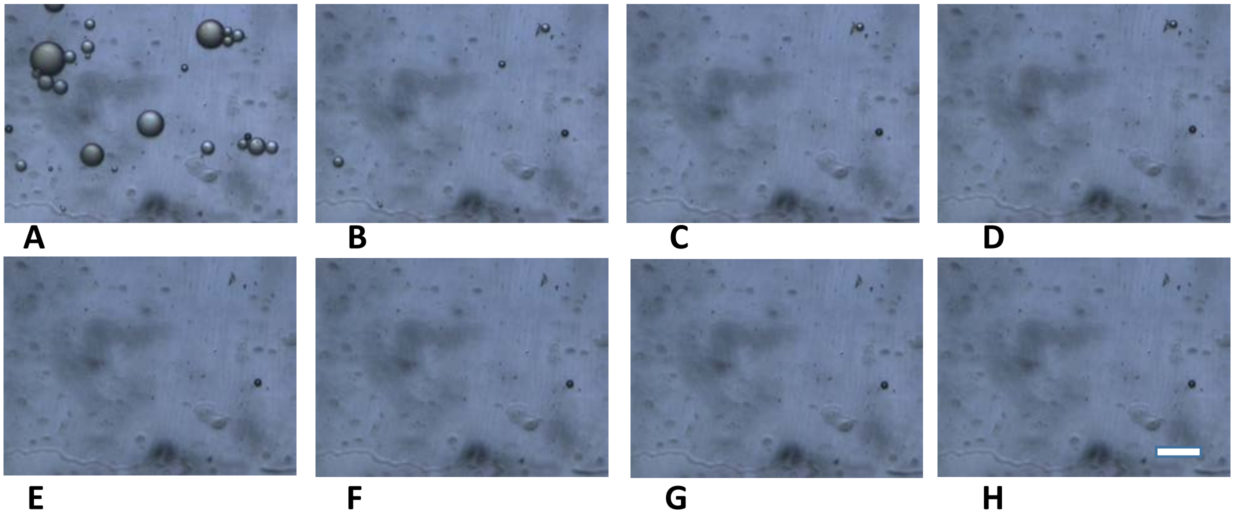

Figure S11. Control experiment. Optical images of the PS beads on the PNIPAM brush surface (37 ^{o}C) A, B, C, D, E, F, G, H, at different wall shear stresses 0.45; 0.93; 1.42; 1.90; 2.39; 2.87; 3.36; 3.84 Pa, respectively. The scale bar is 200 µm.

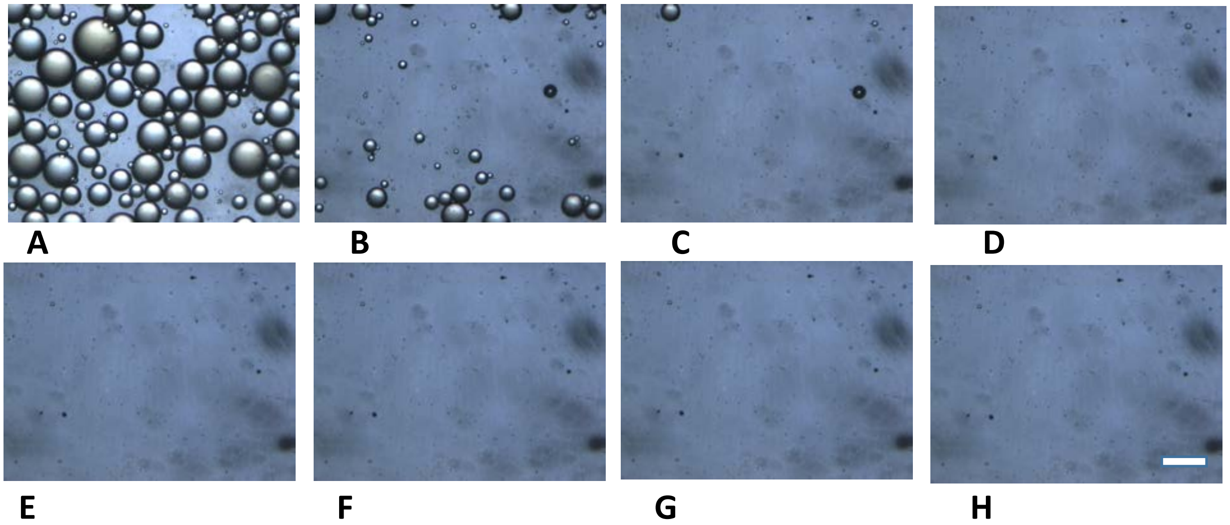

Figure S12. Control experiment. Optical images of the PS beads on the PNIPAM brush surface (25 ^{o}C) A, B, C, D, E, F, G, H, at different wall shear stresses 0.65; 1.35; 2.05; 2.75; 3.45; 4.15; 4.85; 5.55 Pa, respectively. The scale bar is 200 µm.

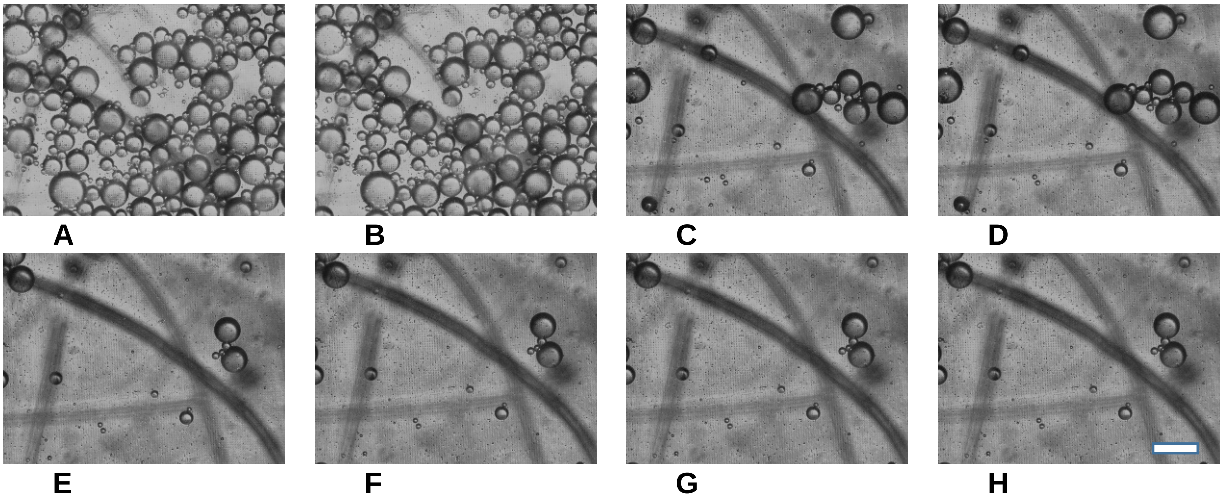

Figure S13. Optical images of the PS beads on the reversible interface sample S2 (h/H = 2.18), 37 ^{o}C: A, B, C, D, E, F, G, H, at different wall shear stresses 0.65; 1.35; 2.05; 2.75; 3.45; 4.15; 4.85; 5.55 Pa, respectively. The scale bar is 200 µm.

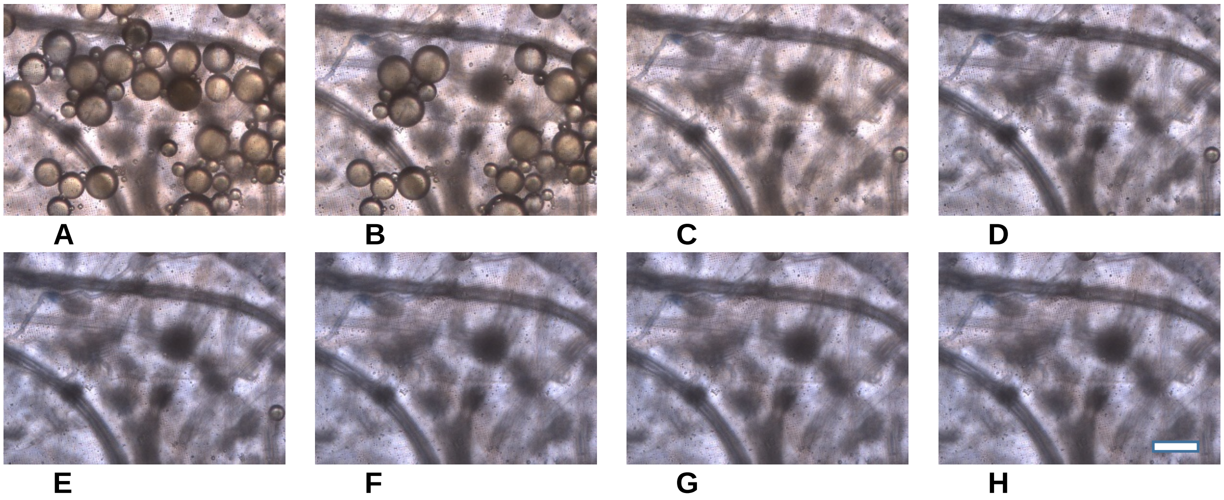

Figure S14. Optical images of the PS beads on the reversible interface sample S2 (h/H = 2.18), 25 ^{o}C: A, B, C, D, E, F, G, H, at different wall shear stresses 0.65; 1.35; 2.05; 2.75; 3.45; 4.15; 4.85; 5.55 Pa, respectively. The scale bar is 200 µm.

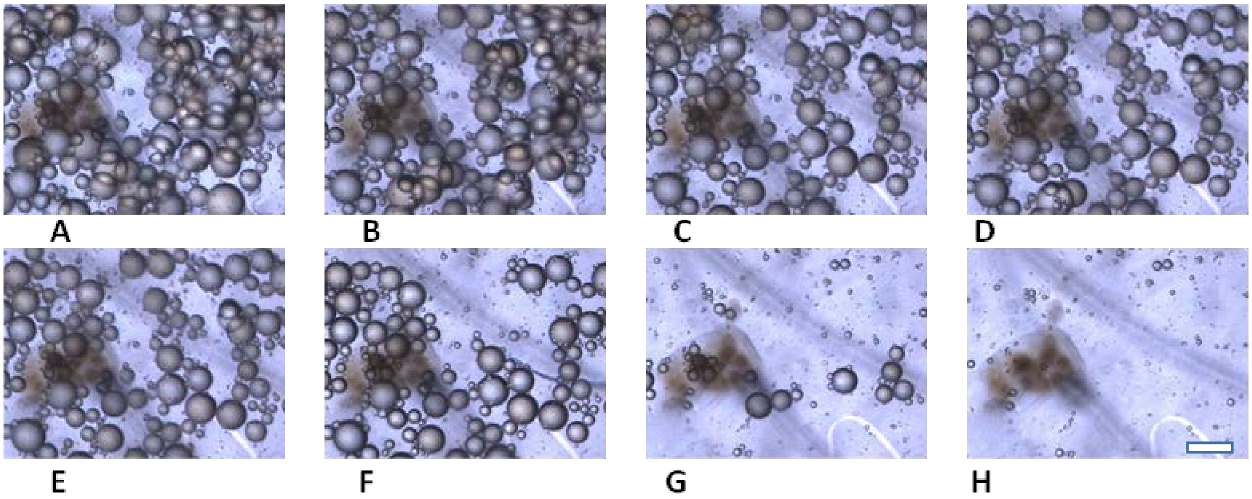

Figure S15. Optical images of the PS beads on the reversible interface, sample S5(h/H = 0.57), 37 ^{o}C: A, B, C, D, E, F, G, H, at different wall shear stresses 0.45; 0.93; 1.42; 1.90; 2.39; 2.87; 3.36; 3.84 Pa, respectively. The scale bar is 200 μm.

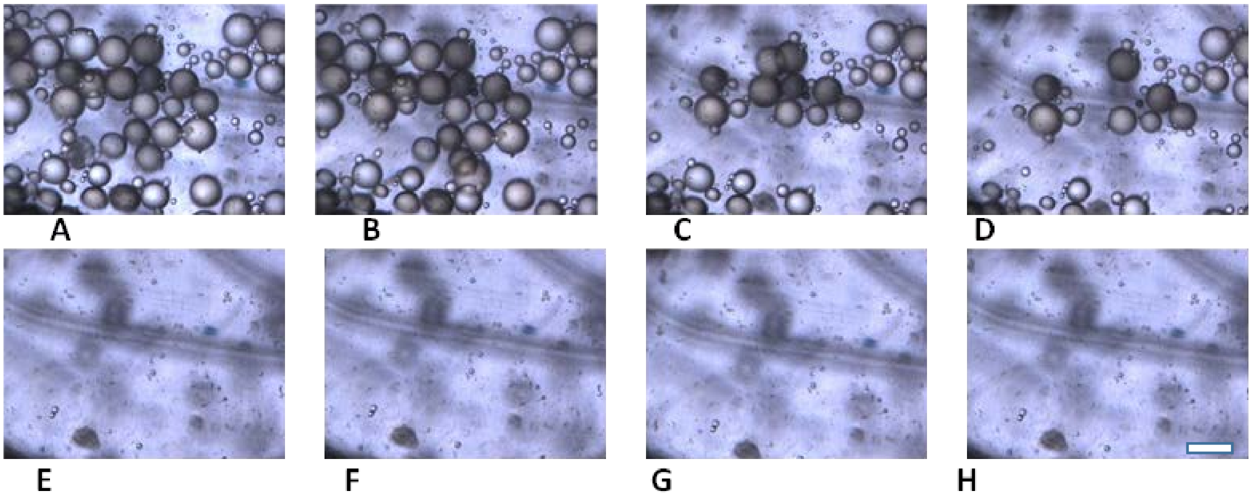

Figure S16. Optical images of the PS beads on the reversible interface sample S5 (h/H = 0.57), 25 ^{o}C: A, B, C, D, E, F, G, H, at different wall shear stresses 0.65; 1.35; 2.05; 2.75; 3.45; 4.15; 4.85; 5.55 Pa, respectively. The scale bar is 200 μm.

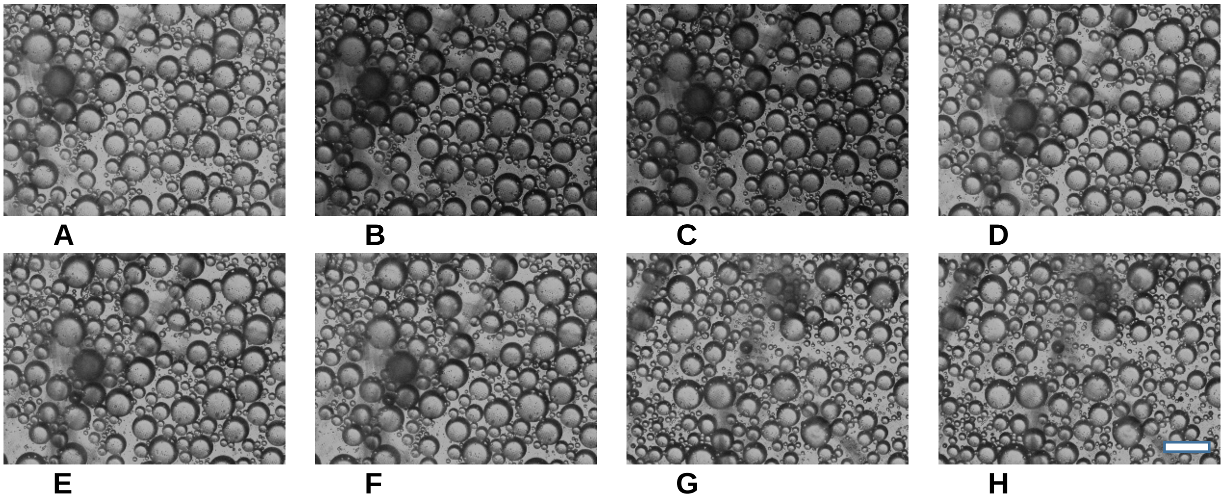

Figure S17. Optical images of the PS beads on the reversible interface sample S6 (h/H = 0.24), 37 °C: A, B, C, D, E, F, G, H, at different wall shear stresses 0.65; 1.35; 2.05; 2.75; 3.45; 4.15; 4.85; 5.55 Pa, respectively. The scale bar is 200 µm.

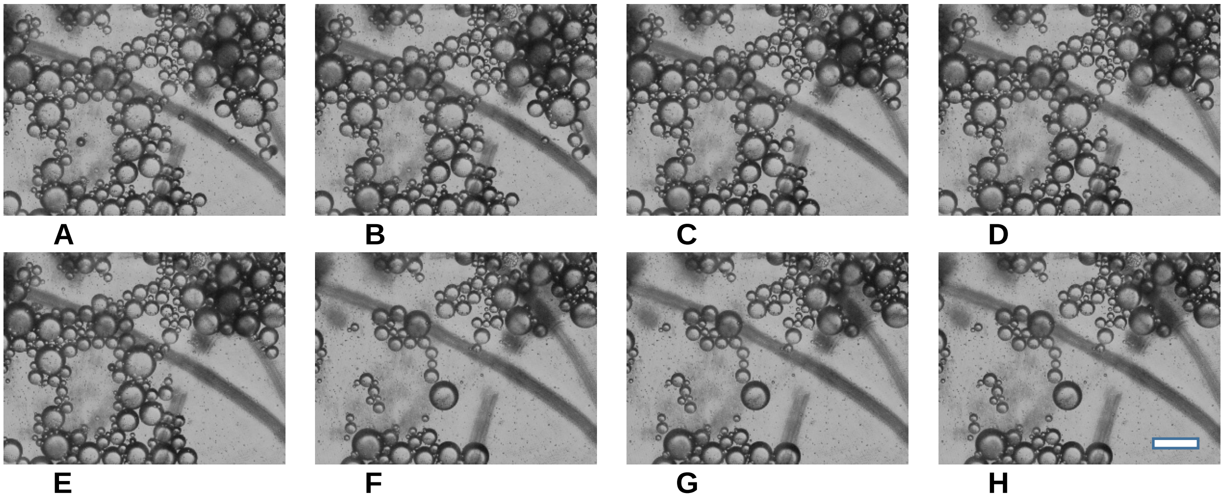

Figure S18. Optical images of the PS beads on the reversible interface sample S6 (h/H = 0.24), 25 °C: A, B, C, D, E, F, G, H, at different wall shear stresses 0.65; 1.35; 2.05; 2.75; 3.45; 4.15; 4.85; 5.55 Pa, respectively. The scale bar is 200 µm.

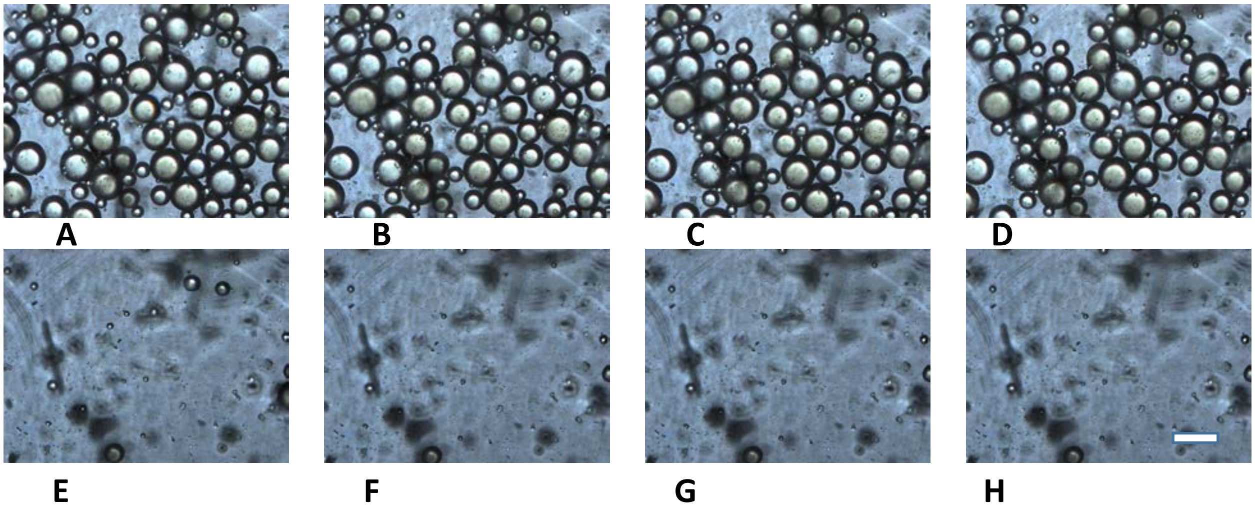

Figure S19. Optical images of the PS beads on the reversible interface sample S7 (h/H = 1.13), 37 °C: A, B, C, D, E, F, G, H, at different wall shear stresses 0.45; 0.93; 1.42; 1.90; 2.39; 2.87; 3.36; 3.84 Pa, respectively. The scale bar is 200 μm.

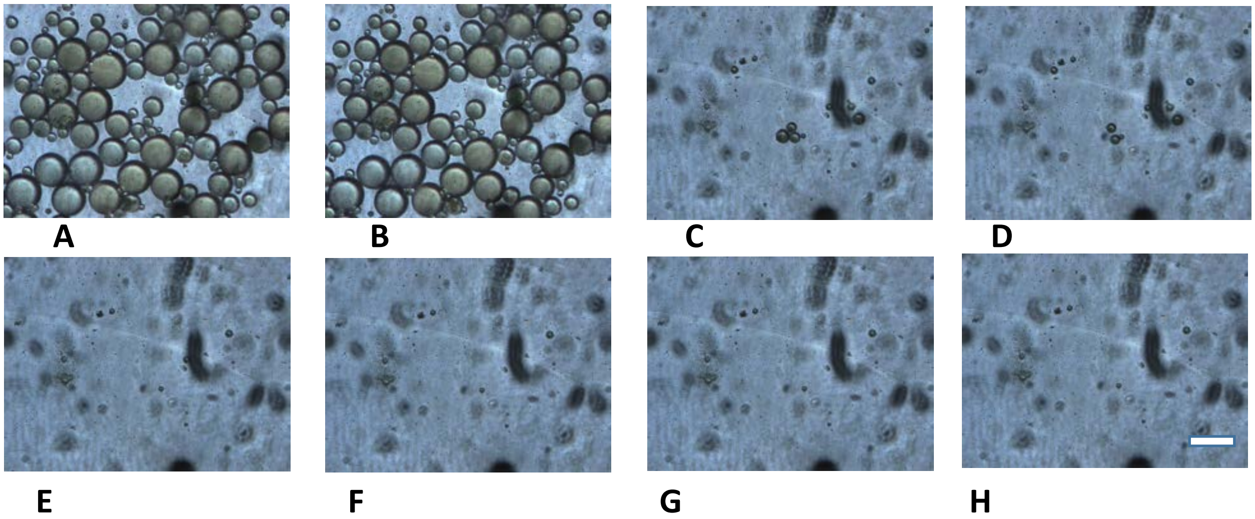

Figure S20. Optical images of the PS beads on the reversible interface sample S7 (h/H = 1.13), 25 °C: A, B, C, D, E, F, G, H, at different wall shear stresses 0.65; 1.35; 2.05; 2.75; 3.45; 4.15; 4.85; 5.55 Pa, respectively. The scale bar is 200 μm.

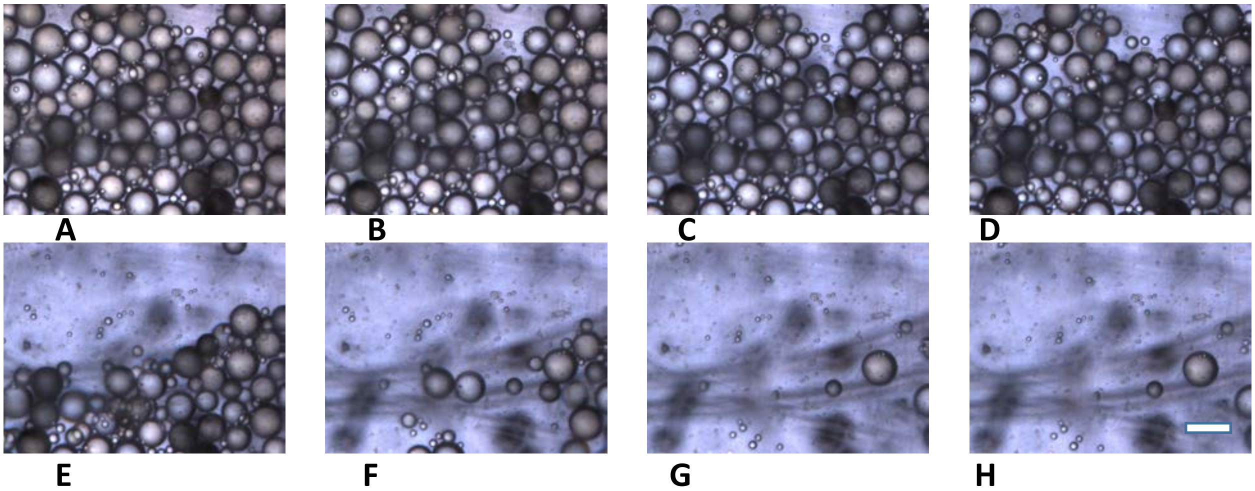

Figure S21. Optical images of the PS beads on the reversible interface sample S9 (h/H = 1.24), 37 ^{o}C: A, B, C, D, E, F, G, H, at different wall shear stresses 0.45; 0.93; 1.42; 1.90; 2.39; 2.87; 3.36; 3.84 Pa, respectively. The scale bar is 200 µm.

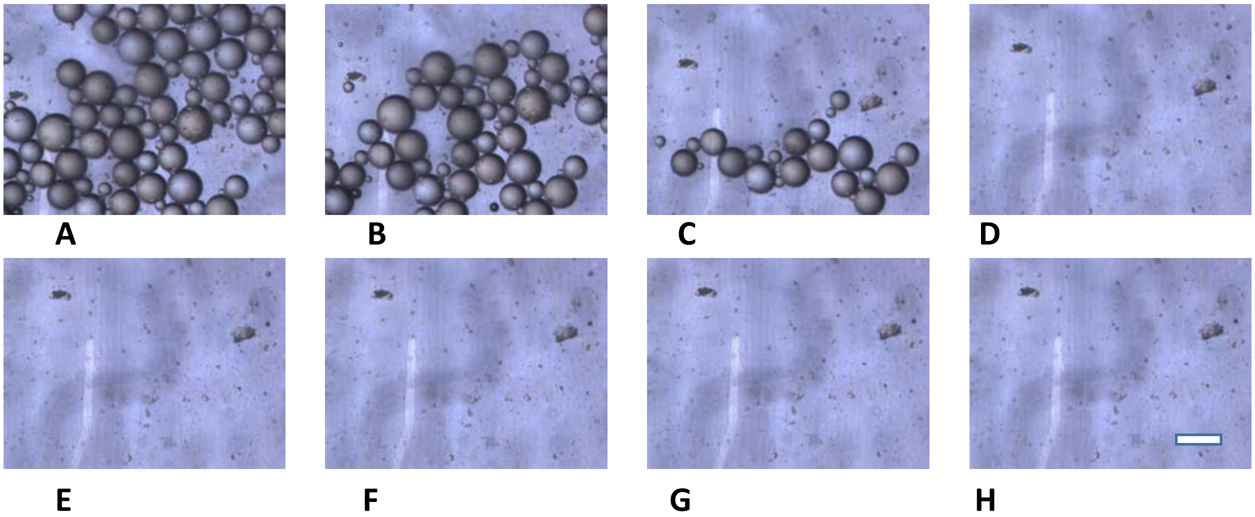

Figure S22. Optical images of the PS beads on the reversible interface sample S9 (h/H = 1.24), 25 ^{o}C: A, B, C, D, E, F, G, H, at different wall shear stresses 0.65; 1.35; 2.05; 2.75; 3.45; 4.15; 4.85; 5.55 Pa, respectively. The scale bar is 200 µm.

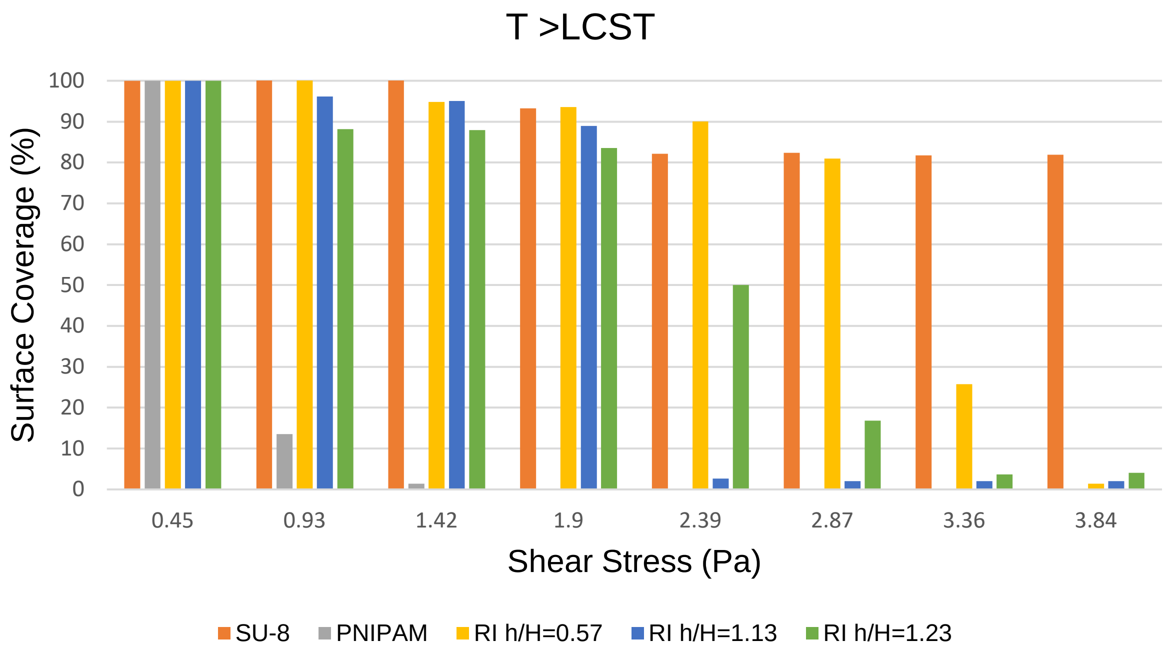

Figure S23. Summary of the experiments with the PS beads, reversible interfaces, and controls at T>LCST. The diagram shows the fraction of the surface coverage by the PS beads at different shear stresses.

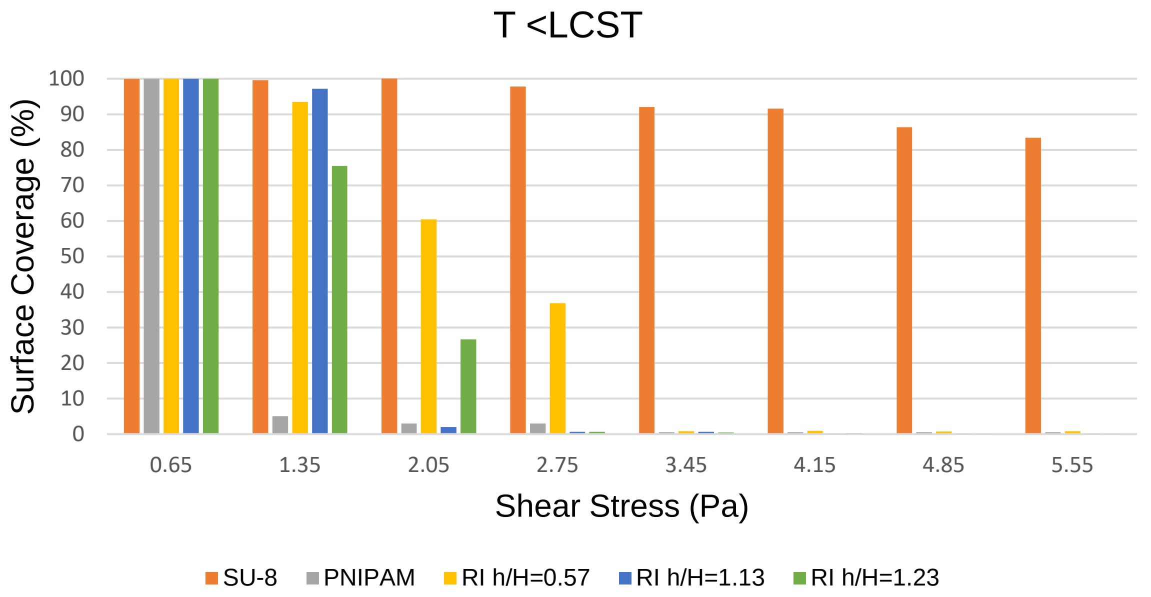

Figure S24. Summary of the experiments with the PS beads, reversible interfaces, and controls at T<LCST. The diagram shows the fraction of the surface coverage by the PS beads at different shear stresses.
